# Biallelic variants in *RYR1* and *STAC3* are predominant causes of King-Denborough Syndrome in an African cohort

**DOI:** 10.1038/s41431-025-01795-z

**Published:** 2025-02-18

**Authors:** Maryke Schoonen, Mahmoud Fassad, Krutik Patel, Michelle Bisschoff, Armand Vorster, Tendai Makwikwi, Ronel Human, Elsa Lubbe, Malebo Nonyane, Barend C. Vorster, Jana Vandrovcova, Michael G. Hanna, Robert W. Taylor, Robert McFarland, Lindsay A. Wilson, Francois H. van der Westhuizen, Izelle Smuts

**Affiliations:** 1 Mitochondria Research Group, Biomedical and Molecular Metabolism Research (BioMMet), North-West University, Potchefstroom, South Africa; 2 Mitochondrial Research Group, Translational and Clinical Research Institute, Faculty of Medical Sciences, Newcastle University, Newcastle upon Tyne, UK; 3 Human Genetics Department, Medical Research Institute, Alexandria University, Alexandria, Egypt; 4 Department of Paediatrics, Steve Biko Academic Hospital, University of Pretoria, Pretoria, South Africa; 5 Laboratory for Inborn Errors of Metabolism (PLIEM), Centre for Human Metabolomics (CHM), Potchefstroom Campus, North-West University, Potchefstroom, South Africa; 6 Centre for Neuromuscular Diseases, UCL Queen Square Institute of Neurology, London, UK; 7 NHS Highly Specialised Service for Rare Mitochondrial Disorders, Newcastle upon Tyne Hospitals NHS Foundation Trust, Newcastle upon Tyne, UK

**Keywords:** Neuromuscular disease, Genetics research

## Abstract

King-Denborough Syndrome (KDS) is a congenital myopathy (CM) characterised by myopathy, dysmorphic features and susceptibility to malignant hyperthermia. The objective of this study was to investigate the genotype-phenotype correlation in Black African patients presenting with CM, specifically those with KDS-like phenotypes, who remained undiagnosed for over 25 years. A cohort of 67 Black African patients with CM was studied, of whom 44 were clinically evaluated and diagnosed with KDS. Whole-exome sequencing (WES) was performed as part of an international genomics study (ICGNMD) to identify potential pathogenic mutations. Genomic assessments focused on identifying relevant genes, including *RYR1* and *STAC3*, and establishing genotype-phenotype correlations. The study identified *RYR1* and *STAC3* mutations as the predominant genetic causes of KDS in this cohort, with mutations in both genes exhibiting autosomal recessive inheritance. While *RYR1* has previously been linked to autosomal dominant mutations, *STAC3*, which was formerly associated exclusively with Native American Myopathy/Bailey-Bloch Myopathy, congenital hypotonia, and susceptibility to malignant hyperthermia, is now newly associated with CM-KDS in this study. This establishes the first genotype-phenotype correlation for 44 Black African individuals with KDS. This study marks a significant milestone in research on understudied African populations with CM, emphasising the lengthy diagnostic journey these patients endured. The findings highlight the pressing need for improved access to genomic medicine in underserved regions and underscore the importance of expanding research and diagnostic capabilities in Africa. This work contributes to the advancement of genetic medicine in underrepresented populations, facilitating better diagnostic and therapeutic outcomes.

## Introduction

King-Denborough syndrome (KDS, OMIM 619542) is a rare form of neuromuscular disorders, more specifically, congenital myopathy (CM) [1, 2]. The clinical spectrum of CM is broad and is typically diagnosed clinically (after extensive neurological evaluations) in neonates presenting with generalised hypotonia, muscle weakness, facial weakness, respiratory insufficiency, and feeding difficulties [3–6]. More specifically, in KDS, patients are characterised by myopathic and facial dysmorphia, musculoskeletal abnormalities, and susceptibility to malignant hyperthermia (MH) [1, 7, 8]. Furthermore, in addition to clinical diagnosis, a comprehensive, precise diagnosis often depends on biochemical evaluations such as muscle imaging, muscle biopsy, and comprehensive genetic testing. Identifying a genetic cause in a patient with a suspected inherited KDS requires access to genetic testing and expert evaluation of results, considering neurological/muscular clinical features, age of onset, inheritance patterns, and population-specific trends. The aetiology of KDS in African patients is largely unknown; however, it has been reported in other populations to have an underlying genetic cause due to autosomal dominant mutations in the *RYR1* gene [2, 9]. Research conducted in South Africa has identified common genetic autosomal recessive inherited *RYR1* mutations [(c.5726_5727delAG (exon 35), c.6175_6187del (exon 38), c.8342_8343delTA (exon 53), c.14524G>A (exon101), and c.10348-6C>G (intron 68)] in patients with CM characterised by central nuclei myopathy, with no clinical characteristics of KDS [10]. The presence of *STAC3* pathogenic c.815G>C; p.Trp284Ser homozygous variant has been reported in other groups with African ancestry [11, 12], and in 2024, a South African study confirmed homozygous *STAC3*:c.851C>G in 25 out of 127 (20%) participants presenting with congenital hypotonia [13]. Prior to this, we identified the first homozygous *STAC3*:c.851G>C variant in a Black African female patient as part of a study investigating the potential nuclear genetic background of patients with biochemically confirmed mitochondrial disorders [14].

Our paediatric neurology clinic at Steve Biko Academic Hospital (SBAH) in Pretoria, provides tertiary care services, primarily serving the northern regions of South Africa. Over the past 25 years, we have observed a subset of patients with CM who present with clinical and phenotypic features strikingly similar to those of KDS. These patients exhibit a range of KDS characteristics, including myopathy, ptosis, malar hypoplasia, skeletal and other dysmorphic features, cryptorchidism, and, in some cases, documented episodes of MH following surgery. The recurring presence of these prominent features strongly suggests a shared underlying genetic aetiology in this patient cohort with a KDS-like phenotype.

In this study, we used whole exome sequencing (WES)—accessed through the International Centre for Genomic Medicine in Neuromuscular Diseases (ICGNMD)—to investigate a cohort of 67 South African participants in the ICGNMD study with neuromuscular diseases of suspected genetic origin. We identified a genetic diagnosis for 54 CM participants (~81%), 44 of which presented with KDS-like features. Notably, all participants with KDS-like features were found to carry biallelic variants in the *RYR1* and *STAC3* genes, with the latter representing a novel genetic association with KDS. These findings highlight the importance of *RYR1* and *STAC3* as common genetic causes of neuromuscular diseases in African patients. Our results advocate for routine screening of these genes in patients presenting with KDS-like features to improve diagnosis and treatment.

## Patients and methods

### Study recruitment and inclusion/exclusion criteria

Ethical clearance was granted (Approval Numbers: 296/2019 from the University of Pretoria and NWU-00966-19-A1 from the North-West University) to recruit patients with a suspected inherited NMD as well as their affected or unaffected relatives for clinical and genetic analyses. Three central hospitals in Gauteng provide specialised services for NMD. There are ~8 million children between 0 and 14 years of age in Gauteng and the four neighbouring provinces (https://www.statssa.gov.za/ last accessed June 2024). Although there is a slight overlap in the referral areas of the three facilities, Steve Biko Academic Hospital (SBAH) predominantly provides services to the northern municipal regions in Gauteng, as well as the Limpopo and Mpumalanga provinces with a paediatric population of approximately 4 million between the ages of 0 and 14 years. The recruitment site manages the entire spectrum of paediatric neurology and neurodevelopmental disorders, with an average of 6500 patients per annum.

All individuals with a clinical phenotype compatible with NMD of a suspected genetic origin were eligible for inclusion in the broader ICGNMD project. Here, we report on the subset of participants with onset of symptoms at birth or within the first six months of life (the congenital group) and with completed genetic analysis (WES, if initial local investigations did not confirm a diagnosis). In addition, participants with muscle weakness not explained by known phenotypes as described in the exclusion criteria were also included.

Exclusion criteria comprised incomplete genetic information and patients with a prior diagnosis of Duchenne/Becker muscular dystrophy, metabolic myopathy, spinal muscular atrophy or other syndromes associated with neonatal hypotonia and/or muscle weakness.

**Fig. 1:**
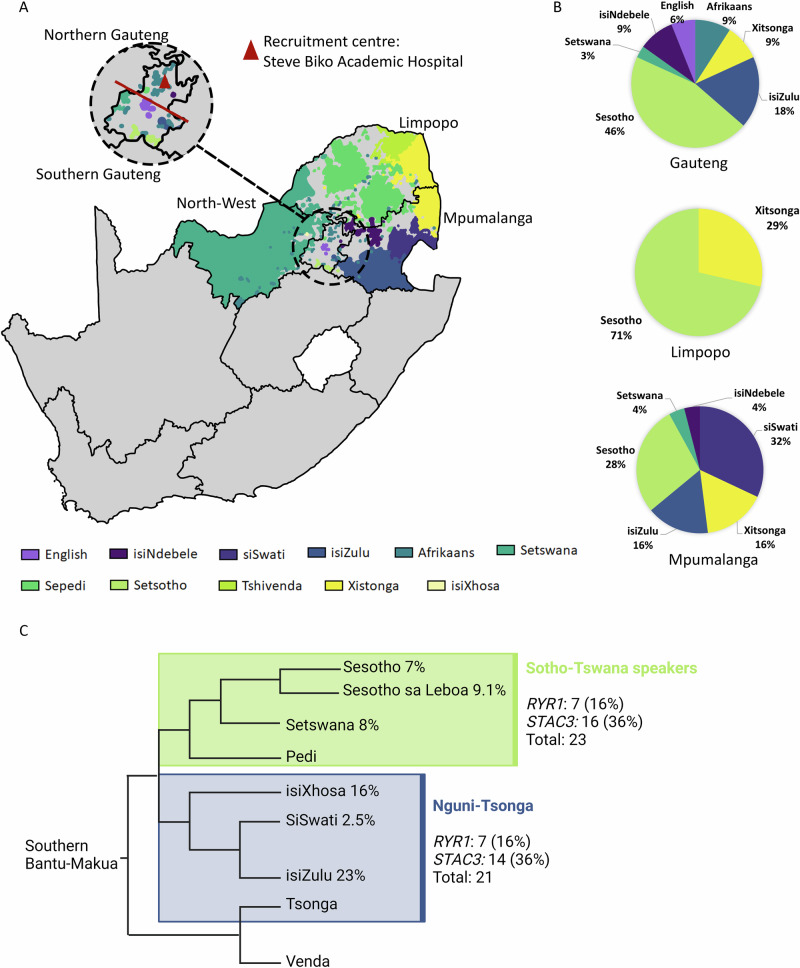
Ethno-linguistic distribution in South Africa. **A** Geographical distributions of first or home languages in South Africa, illustrating linguistic diversity in the northern provinces: Limpopo, Mpumalanga, Gauteng, and North-West Province. **B** Pie charts showing the distribution of the 67 study participants according to their primary language, broken down by referring province. **C** Language group distribution of 44 KDS-like participants (with *RYR1* and *STAC3* mutations), categorised by major language groups: Nguni-Tsonga or Sotho-Tswana.

Between October 2020 and February 2024, 255 probands and 377 family members (23 clinically affected) gave informed consent to participate in the ICGNMD study. Participants were mainly recruited from the northern provinces of South Africa (Fig. 1A–C).

To assess the ethno-linguistic influence on the disease patterns, the ethnicity, language group, self-reported language, and province of origin were collected. There are nine provinces, 12 official languages, and four major population groups in South Africa. The latter are officially referred to as Black African, Coloured/mixed, Asian/Indian, and White.

The nine major African languages originally stem from the Niger-Congo family. Within this family, the Southern Bantu-Makua group is divided into the Nguni-Tsonga and Sotho-Makua-Venda groups, which are further subdivided into the Sotho-Tswana and Tshivenda groups. These ethno-linguistic groups are spread across the country, with different languages dominating various provinces.

### Participant assessment and data collection

A thorough clinical assessment was conducted for all probands and affected relatives. Pseudonymised participant data were entered into the ICGNMD-REDCap Study Database, which includes the family history, relevant medical and surgical history, clinical findings, and results of available prior investigations. Human Phenotype Ontology (HPO) terms were used to summarise the key clinical features for maximum interoperability (https://hpo.jax.org/app/). Demographic details and medical histories were documented, including pedigrees (Supplementary Fig. S1).

### Participant sample collection

Whole blood samples (5 ml) from consenting participants were collected and stored (−80 °C) according to standard procedure until DNA extraction was performed using the FlexiGene® DNA Kit (51206; Qiagen Science, Hilden, Germany).

### Genetic testing and whole-exome sequencing (WES) data analysis

All participants, except two with locally confirmed diagnoses, underwent WES according to the protocol described by Wilson et al. [15]. The relevant CM and CD panels (Genomics England PanelApp, v.4.38 and v.4.24, https://panelapp.genomicsengland.co.uk, last accessed July 2024) were applied. Variants of interest were classified according to the American College of Medical Genetics and Genomics (ACMG) guidelines [16]. Potential disease-causing variants were validated and examined for segregation via familial Sanger sequencing at the North-West University, Potchefstroom Campus, South Africa. Sanger data were analysed using GEAR-Genomics (https://www.gear-genomics.com, last accessed June 2024) [17].

### STAC3-variant haplotyping

To assess the homozygosity and potential for founder effects within the region of the *STAC3:*c.851G>C variant, a subset of 26 samples was analysed according to the protocol described in Bisschoff et al. [18]. Analysis was hampered by the under-representation of Black South African sub-population allele frequency data in linkage disequilibrium and Maximum Allele Frequency data in publicly available datasets, with the “geographically/genetically closest” but distant, being that of the ‘Yoruba in Ibadan, Nigeria’ population data from the 1000 Genomes Project. *RYR1* haplotyping was previously documented for the local population from the southern regions of South Africa by Wilmshurst et al. [10].

### Population analysis and frequency determination for the STAC3:c.851G>C variant

Ethics approval (NWU-00966-19-A1) included population-based screening for the most common variant detected in the cohort, *STAC3*(NM_145064.3):c.851G>C, using PCR-RFLP analysis. DBS cards, collected between 2020 and 2022 from healthy newborns of parents from the northern provinces and belonging to the four largest population groups in South Africa, were used as a DNA source. The gender distribution of the samples was equal, and all samples were anonymised and randomised, with 750 available for each ethnicity apart from the Coloured/mixed group, where only 594 samples could be sourced. The DBS cards were collected for routine metabolic testing (not due to suspicion of a metabolic disorder) and were considered representative of the local populations.

Briefly, DNA was amplified directly from the DBS cards Phire HS II Master Mix (F170L, Thermo Scientific, Massachusetts, USA) and the following primers: Forward 5′-TTA ATG GTT AAG CTC TCC AAG GAG TGT-3′, Reverse 5′-GTA CTG CGG AGT GAA GAG GAA AGA-3′. The region spanning the *STAC3*:c.851G>C variant was then digested by the restriction enzyme *TfiI* (R0546S, New England Biolabs, Massachusetts, USA), which recognises and cuts the variant sequence (5′-G^AWTC-3′). Frequencies were calculated and stratified by broad-reported ethnic group using the formula:

$${{\rm{p}}}={{\rm{f}}}\left({{\rm{AA}}}\right)+1/2\;{{\rm{f}}}\left({{\rm{AB}}}\right)$$

Where p represents the allele frequency of the variant, AA is the ratio of homozygous affected samples per total number of samples per population group, and AB is the ratio of heterozygous samples per total number of samples per population group.

The *STAC3* frequency was then compared to other population groups listed in gnomAD [African/African American and European (Non-Finnish), v4.1.0, https://gnomad.broadinstitute.org/ last accessed July 2024] and the Human Heredity and Health in Africa (H3Africa) project (African, Caucasian and Coloured/mixed) https://agvd-dev.h3abionet.org, last accessed July 2024).

## Results

### Demographics

**Fig. 2:**
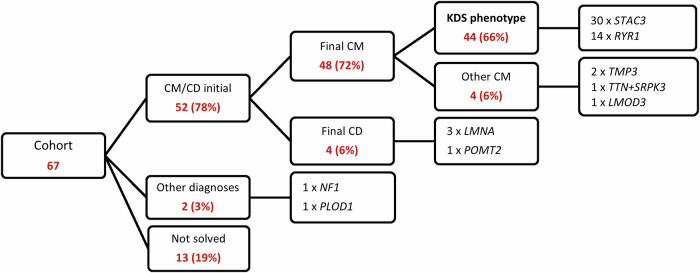
Summary of the overall phenotypic and genetic findings for the original neuromuscular disease cohort of 67 participants.

Figure 1A–C summarises the geographic and ethno-linguistic distribution of the KDS cohort in South Africa. The majority of participants, 56 (84%), came from our direct referral areas, which include Northern Gauteng and the Limpopo, and Mpumalanga provinces. The remaining 11 (16%) were referred to our clinic for specialised opinions and diagnostic purposes. Sixty-seven participants with a broad clinical phenotype of CM/CD met the sub-study inclusion criteria. The overall outcomes of the entire cohort are shown in Fig. 2. Following WES analysis, the genetic diagnosis of 13 (19%) participants remained unsolved. Alternative diagnoses of Neurofibromatosis (*NF1*-variant) and Ehlers-Danlos syndrome (*PLOD1*-variant) were identified in two participants. Out of the 52 remaining participants, four had a final diagnosis of CD and 48 of CM. Four of the 48 CM participants had a genetic diagnosis other than *STAC3*- or *RYR1*-variants, while the remaining 44 participants, all displaying the clinical features suggestive of KDS, carried either *STAC3-* or *RYR1-*variants.

### Clinical and biochemistry findings

**Fig. 3:**
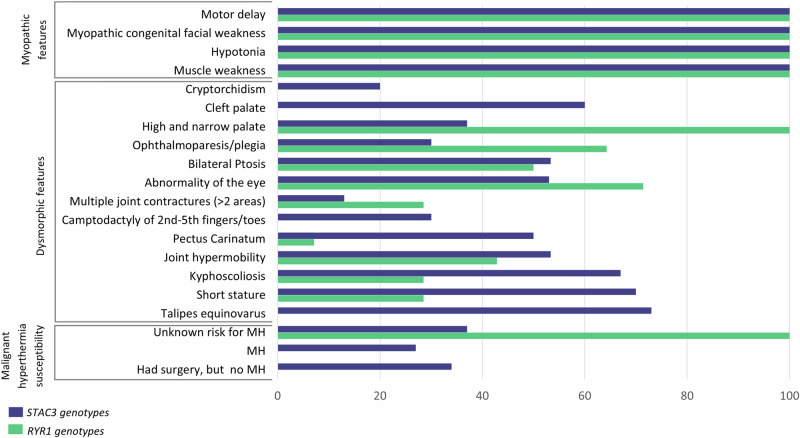
Comparison of the most prominent clinical features, based on the three King-Denborough syndrome classifications, for the *RYR1* and *STAC3* genotypes in participants with KDS. All values are expressed as percentages.

The distinctive clinical features of the 52 participants with CM/CD phenotype are shown in Table S1 and Fig. 3. As expected, the outstanding difference between the genetically diagnosed CD and CM groups was the markedly higher CK values observed for the former (mean value 2060 IU/L vs 96 IU/L, *p* < 0.001). In the CM group, three of the four participants (75%) with a genetic diagnosis other than *RYR1* or *STAC3* had marked neck flexor weakness. This was not recorded in any of the KDS-like participants. The 44 participants make up the KDS-like cohort are described in greater detail below.

### Included families

The group spanned 42 families and included 23 males and 21 females. Eighteen (40%) participants had a history of affected siblings, two of whom were included in the study. The pedigrees and relevant segregation analyses are summarised in Fig. S1.

### Perinatal history

Twenty-five (57%) participants were reported to have experienced decreased foetal movement, and five (11%) were in breech presentation. All participants were floppy at birth, with only 4 (9%) not experiencing feeding difficulties. Respiratory difficulties were reported in 22 (50%) babies, although none required ventilatory support during the neonatal period.

### Myopathy

All participants exhibited hypotonia, generalised muscle weakness, and facial weakness (myopathic facies) since birth. The facial weakness aligned with the description of Webb et al. [19] and associated with an expressionless face, absence of nasolabial and periorbital folds, incomplete eyelid closure, open triangular mouth, and drooling. Older participants also exhibited articulation difficulties and peri-oral weakness [19].

### Dysmorphic features

Eye abnormalities were documented in 26 (59%) participants. Bilateral ptosis was present in approximately half of the participants in each group: 7 (50%) in the *RYR1* group and 16 (53%) in the *STAC3* group. External ophthalmoplegia was observed in 9 (64%) of the *RYR1* group but only in 5 (17%) of the *STAC3* group. A high prevalence of external ophthalmoplegia was also reported in another South African cohort with *RYR1* variants, as noted by Wilmshurts et al. [10].

All participants had palatal abnormalities. In the *STAC3* group, the majority, (19 or 63%) had cleft palates, while the remaining 11 (37%) had high, narrow palates. All 14 (100%) participants in the *RYR1* group had high, narrow palates.

Although neck abnormalities are documented in KDS [2], they were not prevalent in our cohort. Only three (10%) participants in the S*TAC3* group exhibited short necks based on clinical assessment. In the *STAC3* group, 22 (50%) presented with talipes equinovarus, nine (30%) with camptodactyly and six (20%) with cryptorchidism. None of these phenotypes was observed in the *RYR1* group. Joint hypermobility was present in 22 (50%) KDS-like participants. Multiple contractures were more evident in the *RYR1* group [4 (29%)] than in the *STAC3* group [4 (13%)]. Pectus carinatum and kyphoscoliosis were proportionally more prevalent in the *STAC3* group [15 (50%) and 20 (67%)] compared to the *RYR1* group [1 (7%) and 4 (29%)]. Interestingly, short stature was more prominent in the *STAC3* group [21 (70%)], whilst only reported in 4 (29%) participants in *the RYR1* group. It should be noted that the severity of spinal deformities, such as kyphoscoliosis, can affect the accuracy of height measurements.

### Malignant hyperthermia susceptibility (MHS)

Reliable data on MH are challenging to collect since this is only expected to develop upon administration of volatile anaesthetic gasses (isoflurane) or succinylcholine [20]. The in vitro contracture test has excellent specificity and sensitivity to detect MHS, but is not routinely available nor is it available at our facility [6]. When surgical intervention is necessary for a patient with a CM (e.g., to correct a cleft palate), preventative protocols can be implemented to ameliorate the risk of MH. Eighteen (41%) participants had undergone surgery. Among this subset, MH was observed in eight (18%) participants from the *STAC3* group. An additional 10 (33%) participants in the *STAC3* group underwent surgery with precautionary measures for MHS in place, resulting in an uneventful intra-operative course. None of the participants in the *RYR1* group were exposed to anaesthetics during the period of data collection. Therefore, MHS status, remains unknown for 26 (59%) participants in the entire KDS group.

### Other clinical associations

All 44 participants had areflexia. Four (13%) participants had conductive hearing impairment in the *STAC3* group.

### Genetic findings

As summarised in Table 1, all 44 KDS-like cohort participants were found to have either biallelic *STAC3* or biallelic *RYR1* variants as the likely cause of their CM. No *CACNA1S* variants were detected by WES. Details concerning the other genetic variants identified in the larger CM cohort, are also provided in Table 1.

**Table 1:** Genotypes identified in the CM/CD participants.

Genotype	Allele call (inheritance pattern)	Affected participants	ACMG classification^b^		gnomAD allele frequency^c^
*RYR1*(NM_000540.3):c.[10348-6C>G;14524G>A]; p.[intron68;Val4842Met]^a^ [10, 26]	Compound heterozygous (AR)	P01-P08	LP	PM2 PM3 PP1 PP3 PS3	0.03% 0.02%
*RYR1*(NM_000540.3):c.8342_8343delTA; p.Ile2781Argfs^c^ 49^a^ [10, 26]			P	PVS1 PM2 PM3 PP1	0.02%
*RYR1*(NM_000540.3):c.[10348-6C>G;14524G>A]; p.[intron68;Val4842Met]^a^ [10, 26]	Compound heterozygous (AR)	P09	LP	PM2 PM3 PP1 PP3 PS3	0.03% 0.02%
*RYR1*(NM_000540.3):c.12625-1G>A [10, 26]			LP	PVS1 PM2	<0.001%
*RYR1*(NM_000540.3):c.[10348-6C>G;14524G>A]; p.[intron68;Val4842Met]^a^ [10, 26]	Compound heterozygous (AR)	P10-P11	LP	PM2 PM3 PP1 PP3 PS3	0.03% 0.02%
*RYR1*(NM_000540.3):c.6797-1G>A [10, 26]			P	PVS1 PM2 PP5	0.01%
*RYR1*(NM_000540.3):c.[10348-6C>G;14524G>A]; p.[intron68;Val4842Met]^a^ [10, 26]	Compound heterozygous (AR)	P12	LP	PM2 PM3 PP1 PP3 PS3	0.03% 0.02%
*RYR1*(NM_000540.3):c.2870+1G>T^d^			P	PS4 PVS1 PM2	Absent
*RYR1*(NM_000540.3):c.[10348-6C>G;14524G>A]; p.[intron68;Val4842Met]^a^ [10, 26]	Compound heterozygous (AR)	P13	LP	PM2 PM3 PP1 PP3 PS3	0.03% 0.02%
*RYR1*(NM_000540.3):c.12814_12815insCGCGGAGT p.Glu4273Argfs^c^71^d^ [10, 26]			LP	PVS1 PM2	Absent
*RYR1*(NM_000540.3):c.10280C>T; p.Pro3427Leu	Compound heterozygous (AR)	P14	VUS	PM2	0.01%
*RYR1*(NM_000540.3):c.2682+4A>G^d^			VUS	PM2 PP3	Absent
*STAC3*(NM_145064.3):c.851G>C; p.Trp284Ser [11, 12, 24, 28]	Homozygous (AR)	P15-P42	P	PM3 PP1 PS3	0.1%
*STAC3*(NM_145064.3):c.851G>C; p.Trp284Ser [11, 12, 24, 28]	Compound heterozygous (AR)	P43-P44	P	PM3 PP1 PS3	0.1%
*STAC3*(NM_145064.3):c.834_836del; p.Asp279del^d^			VUS	PM2 PM4	Absent
*LMOD3*(NM_198271.5):c.506C>T; p.Thr169Met	Compound heterozygous (AR)	P45	VUS	-	0.07%
*LMOD3*(NM_198271.5):c.920G>A; p.Arg307His			VUS	PM2	0.004%
*SRPK3*(NM_014370.4):c.260G>A; p.Trp87^c^ [29]	Homozygous (AR)	P46	P	PM2	Absent
*TTN*(NM_001267550.2):c.68389C>T; p.Pro22797Ser [29]	Heterozygous	P46	P	PM2	Absent
*TPM3*(NM_152263.4):c.502C>T; p.Arg168Cys [30]	Heterozygous (AD)	P47	P	PS2 PS3 PM1 PM2 PM5 PP2 PP3	Absent
*TPM3*(NM_152263.4):c.734G>C; p.Arg245Thr^d^ [31]	Heterozygous (AD)	P48	LP	PM2 PM5 PP2 PP3	Absent
*LMNA*(NM_170707.4):c.1095_1112del; p.Ile365_Asp370del^d^	Heterozygous (AD, De novo)	P49	LP	PM1 PM2 PM4	Absent
*LMNA*(NM_170707.4):c.91G>A; p.Glu31Lys [32]	Heterozygous (AD, De novo)	P50	P	PS2 PM1 PM2 PM5 PP2	<0.001%
*LMNA*(NM_170707.4):c.746G>A; p.Arg249Gln [16, 33]	Heterozygous (AD, De novo)	P51	P	PS3 PS4 PM1 PM2 PM5 PP2 PP3	Absent
*POMT2*(NM_013382.7):c.2206del; p.Gln736Lysfs^c^7	Homozygous (AR)	P52	P	PVS1 PM2 PM3	<0.001%

*AD* autosomal dominant, *AR* autosomal recessive, *P* *followed by a number* participant number, *P* pathogenic, *LP* likely pathogenic, *VUS* variant of uncertain significance.

^a^Known Black African founder, for which routine testing is available in South Africa.

^b^For the full list of descriptions, see Richards, Aziz [12].

^c^Based on African genetic ancestry group.

^d^Novel variants.

### RYR1 variants

None of the 44 participants exhibited an AD inheritance pattern as initially described for KDS-associated *RYR1* variants [2, 8, 21]. A total of 14 (32%) participants were found to have compound heterozygous *RYR1* variants, with the majority [13 (93%)] harbouring the known African *RYR1* common allelic combination, *RYR1*(NM_000540.3):c.[10348-6C>G;14524G>A], combined with a splice-altering, in-frame deletion and/or frameshift variant.

### STAC3 variants

Thirty of the 44 participants (68%) carried the pathogenic missense variant, *STAC3*(NM_145064.3):c.851G>C. Among these, 27 (90%) were homozygous for the variant, while three (10%) were compound heterozygous, carrying both the *STAC3*:c.851G>C variant and the novel heterozygous deletion, *STAC3*(NM_145064.3):c.834_836del.

### Cohort comparison of previous studies with RYR1 and STAC3 genotypes

The clinical features of the 44 participants were compared to previously published cohorts (Table 2 and 3), three with *RYR1* genotypes and three cohorts with *STAC3* genotype [2, 9–11, 13, 22]. The most common features observed in our cohort were consistent with CM, including hypotonia (100%), muscle weakness (100%), and respiratory symptoms (100%). Notably, cleft palate (40%), talipes equinovarus (50%), and cryptorchidism (16%) were frequently observed, with a higher incidence than in some other cohorts, reinforcing the unique phenotypic characteristics of KDS-like presentations in African patients.

**Table 2:** Comparison of *RYR1* and *STAC3* genotype and phenotype findings across cohorts with KDS-like features.

		RYR1 genotype			STAC3 gentotype		
	This cohort investigation	Wilmshurst et al. [10]	Dowling et al. [2]	Vasco et al. [9]	Zaharieva et al. [11]	Campbell et al. [22]	Essop et al. [13]
Participants or cases	44	24, cohort	4, cohort	3, case report	18, cohort	32, cohort	31, cohort
Gene of interest identified	*RYR1* (*n* = 14) *STAC3* (*n* = 30)	*RYR1* (*n* = 17)	*RYR1* (*n* = 3)	*RYR1* (*n* = 3)	*STAC3* (*n* = 18)	*STAC3* (*n* = 1)	*STAC3* (*n* = 25)
Referral hospital or area	SBAH	WC SA, UK, EU	Unknown	DESH	UK	RMMCH, NMCH	NHLS cohort
Gender	29 M, 15 F	9 M, 8 F	3 M, 1 F	1 M, 2 F	11 M, 7 F	16 M, 16 F	19 M, 12 F
Cohort age distribution	0–19 years	2–23 years	6–16 years	4–26 years	0–20 years	NP	0–5 years
Ethnicity	44 AF	4 AF, 8 M/O, 10 C	4 C	3 P	8 AF, 6 ME, 2 AC, 1 CO, 1 SAM	26 AF, 1 C, 4 M/O	31 AF
Inheritance patterns observed	AR	AR suspected	AD	AD	AR	AR	NP
Main Clinical Finding	CM: KDS	CM with central nuclei	CM: KDS	CM: KDS	CM with DF and MHS	Baily-Bloch Myopathy	CM with hypotonia
Genetic investigation	WES, HA, SAAF	Targeted gene screening, HA, PQ	Targeted gene seq	NGS panel for CM	WES, NGS panel	WES	SS, HA, SAAF
Variant interpretations and evaluations	ACMG criteria	N/A	N/A	N/A	N/A	ACMG criteria	NP
Validation and segregation analysis	SS	N/A	N/A	N/A	SS	None	None
Biochemical investigations	NP	MH and IC	MB and IC	NP	MH, Muscle MRI	NP	NP

*AC* Afro/Comorian, *ACMG* American College of Medical Genetics and Genomics, *AD* autosomal dominant, *AF* African, *AR* autosomal recessive, *C* caucasian, *CM* congenital myopathy, *CO* Comorian, *DESH* Divino Espírito Santo Hospital, *DF* dysmorphic features, *EU* Europe, *F* female, *HA* haplotype analysis, *KDS* King-Denborough Syndrome, *M* male, *M/O* mixed/other ancestry, *MB* muscle biopsy, *ME* middle eastern, *MH* muscle histology, *NHLS* National Health Laboratory Services, *NMCH* Nelson Mandela Children’s Hospital, *NP* not performed, *P* Portuguese, *PQ* protein quantification, *RMMCH* Rahima Moosa Mother and Child Hospital, *SA* South Africa, *SAAF* South African allele frequency, *SS* Sanger sequencing, *Seq* sequencing, *SAM* South American, *SBAH* Steve Biko Academic Hospital, *WC* Western Cape, *WES* whole exome sequencing, *UK* United Kingdom.

**Table 3:** Clinical phenotype comparison of KDS-like and CM participants with *RYR1* and *STAC3* variants across different cohorts.

	This cohort	This cohort	Wilmshurst et al. [10]	Dowling et al. [2]	Vasco et al. [9]	This cohort	Zaharieva et al. [11]	Campbell et al. [22]	Essop et al. [13]
	Total (*n* = 44)	*RYR1* (*n* = 14)	*RYR1* (*n* = 17)	*RYR1* (*n* = 3)	*RYR1* (*n* = 3)	STAC3 (*n* = 30)	*STAC3* (*n* = 18)	*STAC3* (*n* = 1)	*STAC3* (*n* = 31)
Decreased foetal movement	**25 (56%)**	**6 (43%)**	11 (65%)	-	-	**19 (63%)**	3 (17%)	-	-
Breech presentation	**5 (11%)**	**3 (21%)**	-	-	1 (33%)	**2 (6%)**	1 (5%)	-	-
Floppy baby	**44 (100%)**	**14 (100%)**	-	-	-	**30 (100%)**	-	-	-
Respiratory symptoms	**44 (100%)**	**14 (100%)**	12 (71%)	-	-	**30 (100%)**	8 (44%)	-	7 (23%)
Feeding difficulties	**40 (90%)**	**11 (79%)**	8 (47%)	-	-	**29 (97%)**	14 (78%)	-	-
Muscle weakness	**44 (100%)**	**14 (100%)**	17 (100%)	4 (100%)	3 (100%)	**30 (100%)**	18 (100%)	-	22 (71%)
Hypotonia	**44 (100%)**	**14 (100%)**	16 (94%)	3 (75%)	1 (33%)	**30 (100%)**	15 (83%)	1 (100%)	31 (100%)
Myopathic congenital facial weakness	**44 (100%)**	**14 (100%)**	-	-	3 (100%)	**30 (100%)**	18 (100%)	1 (100%)	27 (87%)
Motor delay	**44 (100%)**	**14 (100%)**	-	3 (75%)	1 (33%)	**30 (100%)**	17 (94%)	-	4/ (13%)
Abnormality of the eye	**26 (59%)**	**10 (71%)**	16 (94%)	-	3 (100%)	**16 (53%)**	1 (5%)	-	18 (58%)
Bilateral Ptosis/ptosis	**23 (52%)**	**7 (50%)**	7 (41%)	3 (75%)	1 (33%)	**16 (53%)**	14 (78%)	-	16 (52%)
Ophthalmoparesis/plegia	**14 (32%)**	**9 (64%)**	-	-	-	**5 (17%)**	-	-	3 (10%)
High and narrow palate	**24 (55%)**	**14 (100%)**	-	-	1 (33%)	**11 (37%)**	7 (39%)	1 (100%)	16 (52%)
Cleft palate	**18 (40%)**	**0**	-	-	-	**18 (60%)**	5 (28%)	0	11 (36%)
Scaphocephaly	**6 (14%)**	**2 (14%)**	-	-	-	**4 (13%)**	-	-	-
Cryptorchidism	**7 (16%)**	**0**	-	1 (25%)	-	**7 (23%)**	7 (39%)	1 (100%)	7 (23%)
Talipes equinovarus	**22 (50%)**	**0**	-	-	2 (67%)	**22 (73%)**	11 (61%)	-	17 (55%)
Camptodactyly of 2nd–5th fingers/toes	**9 (20%)**	**0**	-	-	-	**9 (30%)**	2 (11%)	-	12 (39%)
Multiple joint contractures (>2 areas)	**8 (19%)**	**4 (29%)**	-	-	-	**4 (13%)**	9 (50%)	-	-
Joint hypermobility	**22 (50%)**	**6 (43%)**	-	3 (75%)	-	**16 (53%)**	7 (39%)	-	4 (13%)
Pectus Carinatum	**17 (39%)**	**1 (7%)**	-	-	2 (67%)	**16 (53%)**	-	-	7 (23%)
Lumbar hyperlordosis	**6 (14%)**	**3 (21%)**	-	3 (75%)	3 (100%)	**3 (10%)**	-	-	-
Kyphoscoliosis	**24 (55%)**	**4 (29%)**	3 (18%)	-	-	**20 (67%)**	7 (39%)	-	3 (10%)
Short stature	**25 (57%)**	**4 (29%)**	-	2 (50%)	1 (33%)	**21 (70%)**	8 (44%)	-	-
Areflexia	**44 (100%)**	**14 (100%)**	-	-	1 (33%)	**30 (100%)**	-	1 (100%)	6 (19%)
Mild hearing impairment	**4 (9%)**	**0**	-	-	-	**9 (30%)**	7 (39%)	-	2 (6%)
Unknown risk for MH	**26 (59%)**	**14 (100%)**	-	N/A	2 (67%)	**12 (40%)**	-	-	13 (42%)
Had surgery, but no MH	**10 (23%)**	**0**	-	4 (100%)	1 (33%)	**10 (33%)**	-	-	14 (45%)
MH	**8 (19%)**	**0**	-	0 (0%)	-	**8 (27%)**	10 (56%)	-	4 (13%)

*MH* malignant hyperthermia.

Bold indicates the values observed in this cohort.

### Haplotype for RYR1 and STAC3 in South Africa

**Fig. 4:**
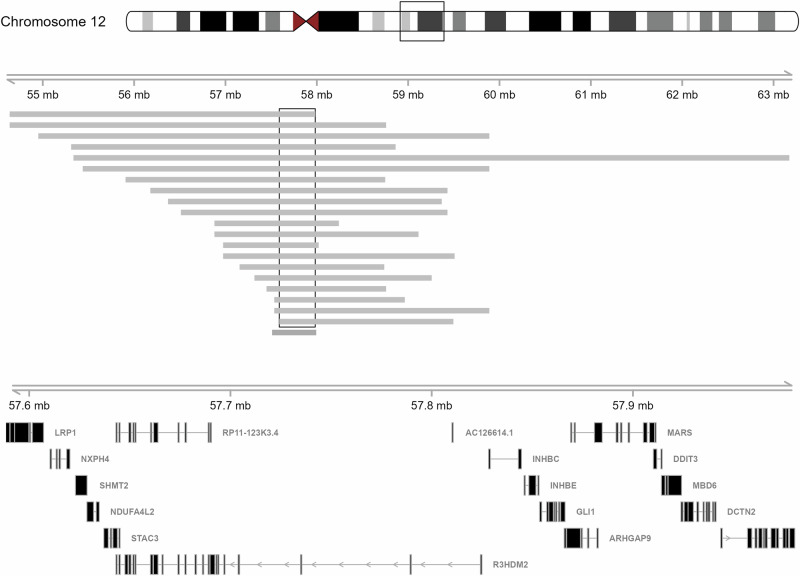
Genotype analysis for a subset of 20 carriers of *STAC3*:c.851G>C.

The WES results confirmed the frequent presence of the *RYR1* haplotype, c.[10348-6C>G;14524G>A], as described by Wilmshurst et al. [10]. The haplotype data for the *STAC3*:c.851G>C, expands on that reported by Essop et al. [13]. We analysed a subset of 26 samples harbouring the homozygous *STAC3*:c.851G>C variant. The results revealed varying lengths of regions of homozygosity, with a minimal shared region of 0.39 Mb, indicating the presence of the same haplotype on both alleles (Fig. 4). These findings suggest the potential emergence of a founder haplotype associated with this *STAC3* variant. However, due to lack of sufficient control data from the same population, accurately estimating the haplotype frequency remains challenging. Additionally, an examination of the Black African data within the 1000 Genomes Project revealed a limited number of single nucleotide polymorphisms (SNPs) in the reported region. These SNPs are characterised by a high heterozygosity rate and low linkage disequilibrium, which further complicates haplotype analysis.

### Allele frequency for STAC3 in South Africa

The allele frequency analysis of 750 healthy DBS samples collected from northern South African provinces for the *STAC3:*c.851G>C variant, revealed a carrier rate of 0.13%. This equates to a disease prevalence of 17.8 cases per 100,000 live births. To validate this finding, we assessed the allele frequency for *STAC3:*c.851G>C, with population frequencies from the gnomAD database (v.4.1, last accessed June 2024), as detailed in Table S2. Importantly, no homozygotes for c.851G>C variant have been identified in the gnomAD population database across any of the listed populations.

## Discussion

The ICGNMD study recruited 632 participants, of which 255 were probands presenting with a neuromuscular disease phenotype, from the northern provinces of South Africa. Of these, 67 were classified as having CM, characterised by features such as hypotonia, muscle weakness and developmental delay. Further investigations revealed that 44 of these 67 CM patients exhibited specific KDS-like phenotypes, comprising a triad of features that includes myopathic and dysmorphic traits, as well as MHS, suggesting an underlying genetic aetiology. Furthermore, all 44 participants were of African descent, representing the largest KDS-like cohort reported from Africa to date, with only two patients previously described in South Africa [23]. The genetic history of KDS reveals an AD inheritance pattern associated with mutations in the *RYR1* gene. In our cohort of 44 KDS-like participants, 14 harboured *RYR1* variants with exclusive AR inheritance, while 30 carried a common *STAC3* variant, also with AR inheritance, previously associated with congenital myopathy and Bailey-Bloch myopathy [11, 24, 25]. This finding suggests a novel genetic aetiology for KDS in African patients.

Distinct phenotypic differences were observed between the *RYR1* and *STAC3* groups (Table S1, Fig. 3 and Tables 2 and 3). Features exclusively associated with the *STAC3*-KDS group included cleft palates, talipes equinovarus, and cryptorchidism (phenotypes observed in KDS).

Additionally, dysmorphic features observed in KDS such as short stature, kyphoscoliosis, and pectus carinatum occurred at a higher frequency in the *STAC3* group, whereas ophthalmoparesis and high narrow palates were more frequent in the *RYR1* group. Notably, none of the *RYR1* participants required surgery during the study period and were classified as having an unknown risk for MH [26].

Furthermore, the phenotypes observed in our cohort align not only with the KDS-like features described in prior studies of *RYR1*-associated disease for KDS but also with broader CM studies focussing on *STAC3-*related variants [2, 9–11, 13, 22]. These studies consistently reported overlapping features of congenital myopathy, dysmorphic traits, and MHS. Notably, the higher frequency of cleft palate, talipes equinovarus, and cryptorchidism in our cohort underscores the distinctive phenotypic contributions of *STAC3* variants to KDS-like presentations. This dual alignment of *STAC3* with both congenital myopathy and KDS phenotypes positions it as a novel finding, expanding the genetic framework of KDS. Furthermore, while *RYR1* and *STAC3* cases from prior studies share many KDS-like features, differences in the prevalence of skeletal dysmorphisms and other traits highlight regional and genetic variability.

The South African haplotype *RYR1*:c.[10348-6C>G;14524G>A], occurring in *cis*, was identified in 13 of the 14 (93%) *RYR1* group. This haplotype was detected as compound heterozygous with five pathogenic or likely pathogenic variants in trans (Table 1). Among these 13 participants (P01-P08), the *RYR1*:c.8324_8326delTA variant, which lies outside an MH hotspot domain, was also present. This deletion affects the domain responsible for binding to DHPR and activating RyR1. Additionally, two splice acceptor variants were identified: *RYR1*:c.12625-1G>A (P09), located in MH hotspot domain 3, and *RYR1*:c.6797-1G>A (siblings P10 and P11), located in MH hotspot domain 2 [26]. Participant P12 carried an intronic splice donor mutation, *RYR1*:c.2780+1G>T, located one base pair downstream of exon 23. This mutation has the potential to interfere with the splicing process of intron 24. Additionally, a significant eight-base pair insertion was detected between positions c.12814 and c.12815 in participant P13. This insertion occurs within the Ca^2+^ binding domain of RyR1, which has a strong affinity for Ca^2+^, and is crucial for activating RyR1. Moreover, this insertion is located within MH domain 3 [26]. Participant P14 had two compound heterozygous variants of uncertain significance (VUS) according to ACMG criteria (Table 1). These variants, *RYR1*:c.10280C>T and *RYR1*:c.2682+4A>G, are located outside of the MH hotspot regions. The former is proposed to be situated in an interdomain interaction region, while the latter is a potential splice variant [26]. When evaluating the severe phenotype of P14, a case can be made to reclassify these variants as likely pathogenic, by adding ‘PP4, Patient’s phenotype or family history is highly specific for a disease with a single genetic aetiology’; however, the severity and penetrance could not be determined.

In this study, we report a novel *STAC3* deletion, *STAC3*(NM_145064.3):c.834_836del; p.Asp279del identified in three unrelated CM participants. This three-nucleotide deletion results in the removal of a single amino acid, aspartic acid at position 279. The variant is in a non-repeat region and has an extremely low frequency in the African population according to gnomAD. This deletion was assigned pathogenic moderate (PM) scores, specifically PM4 and PM2. We propose that this variant, in combination with the known pathogenic *STAC3*:c.851G>C (in trans), likely impacts the functionality of STAC3. These *RYR1* and *STAC3* variants identified in our African participants, are exceedingly rare in global datasets such as gnomAD. To date, there are no reports of the specific African haplotype (*RYR1*:c.[10348-6C>G;14524G>A]) being identified in non-African patients. However, phenotypes associated with *RYR1* and *STAC3* in other populations share significant overlap with those described here, including susceptibility to MH and the triad of KDS features. We suggest that the common *STAC3* variant, c.851G>C, previously identified in many non-African populations could have an African founder effect, given the large region of homozygosity observed in our cohort. Our extensive allele frequency analysis, along with findings from other studies [13], supports a high prevalence (0.13%) of the *STAC3*:c.851G>C variant in the Black African population, especially across the northern provinces of South Africa. Mapping Black African CM participants to ethno-linguistic groups did not reveal significant gene or variant trends within this diverse population. For instance, *RYR1* and *STAC3*-related KDS were present in a 1:2 ratio in both groups (Fig. 1C).

In conclusion, over the last 25 years, patients with CM exhibiting distinctive KDS features, have visited our clinic at Steve Biko Academic Hospital, receiving clinical diagnoses and treatment, but never a genetic confirmation. Through the ICGNMD study initiative [15], we successfully established the genotype–phenotype correlation and provided genetic diagnoses for 44 African KDS-like participants during this study. A genetic diagnosis of *STAC3*-related disease has critical implications for patient care. Many patients with *STAC3*-related disease may require surgeries, such as cleft palate repair or correction of clubfoot, during their lifetime. Identifying pathogenic variants in *STAC3* can inform anaesthetic management, reducing the risk of MH-related morbidity and mortality. This is particularly significant given the high prevalence of MH in *STAC3*-KDS participants in this cohort. A genetic diagnosis also allows for preoperative risk stratification and tailored interventions, underscoring the importance of routine genetic screening in patients with suggestive phenotypes [13, 27]. In South Africa, genetic screening and next-generation sequencing options are limited for the average individual. Given the importance of early detection for treatment, as well as the significant risk of MH during corrective surgical procedures, the National Health Laboratory Services has developed a programme to support low-cost, targeted genetic testing for patients with suggestive phenotypes.

Finally, the study affirms that regional cohorts of CM face diagnostic odysseys that can only be addressed by improving access to genomic medicine and increasing discovery research funding. Emphasising comprehensive history taking and thorough clinical examination, combined with extensive knowledge of associated disease phenotypes and genes, can expedite diagnosis, spare patients from unnecessary and potentially painful investigations, and deliver cost savings.

Relatively low-cost diagnostic algorithms for specific disorders can be developed, given a well-characterised population. Utilising pattern recognition and a stepwise approach in the diagnostic process can resolve most cases, saving both time and money. However, a subset of cases will require a multidimensional approach to fully delineate the underlying pathogenomic mechanisms.

## Supplementary information

Supplementary Table 1

Supplementary Table 2

Supplementary Figure 1

## Data Availability

At the end of the study, participants’ de-identified exome and genome data will be archived in the European Molecular Biology Laboratory European Bioinformatics Institute’s European Genome-Phenome Archive (EMBL EBI EGA), with community access to this and selected de-identified REDCap data managed via an ICGNMD Data Access Committee.
