# Supplementary material for: Biallelic variants in RYR1 and STAC3 are predominant causes of King-Denborough Syndrome in an African cohort
Source: Eur J Hum Genet. 2025 Feb 18;33(4):421–31. doi: 10.1038/s41431-025-01795-z (PMC11985997; doi:10.1038/s41431-025-01795-z)
Supplement: Supplementary file 2 — Supplementary Table 2 [file 41431_2025_1795_MOESM2_ESM.docx]

Table S2: Summary of the *STAC3* allele calls in gnomAD compared to the heterozygous allele calls from northern South African provinces.

|  | **gnomAD populations** | | | | **H3Africa** | | | **This Study: SA Northern Provinces** | | | |
| --- | --- | --- | --- | --- | --- | --- | --- | --- | --- | --- | --- |
| *STAC3*(NM_145064.3):c.851G>C | AF and AM | E | AS | M | AF | E | AS | AF | W and E | I and AS | M |
| Number of heterozygotes | 75 | 9 | 0 | - | 0 | - | - | 2 | 0 | 0 | 0 |
| Allele frequency (%) | 0.09 | 0.0007 | 0 | - | 0 | - | - | 0.13 | 0 | 0 | 0 |

Abbreviations: AF, Black African; AM, African American; AS, Asian; E, European (non-Finnish); I, Indian; M, Mixed ancestry; W, White
