# Supplementary material for: Biallelic variants in RYR1 and STAC3 are predominant causes of King-Denborough Syndrome in an African cohort
Source: Eur J Hum Genet. 2025 Feb 18;33(4):421–31. doi: 10.1038/s41431-025-01795-z (PMC11985997; doi:10.1038/s41431-025-01795-z)
Supplement: Supplementary file 3 — Supplementary Figure 1 [file 41431_2025_1795_MOESM3_ESM.pdf]

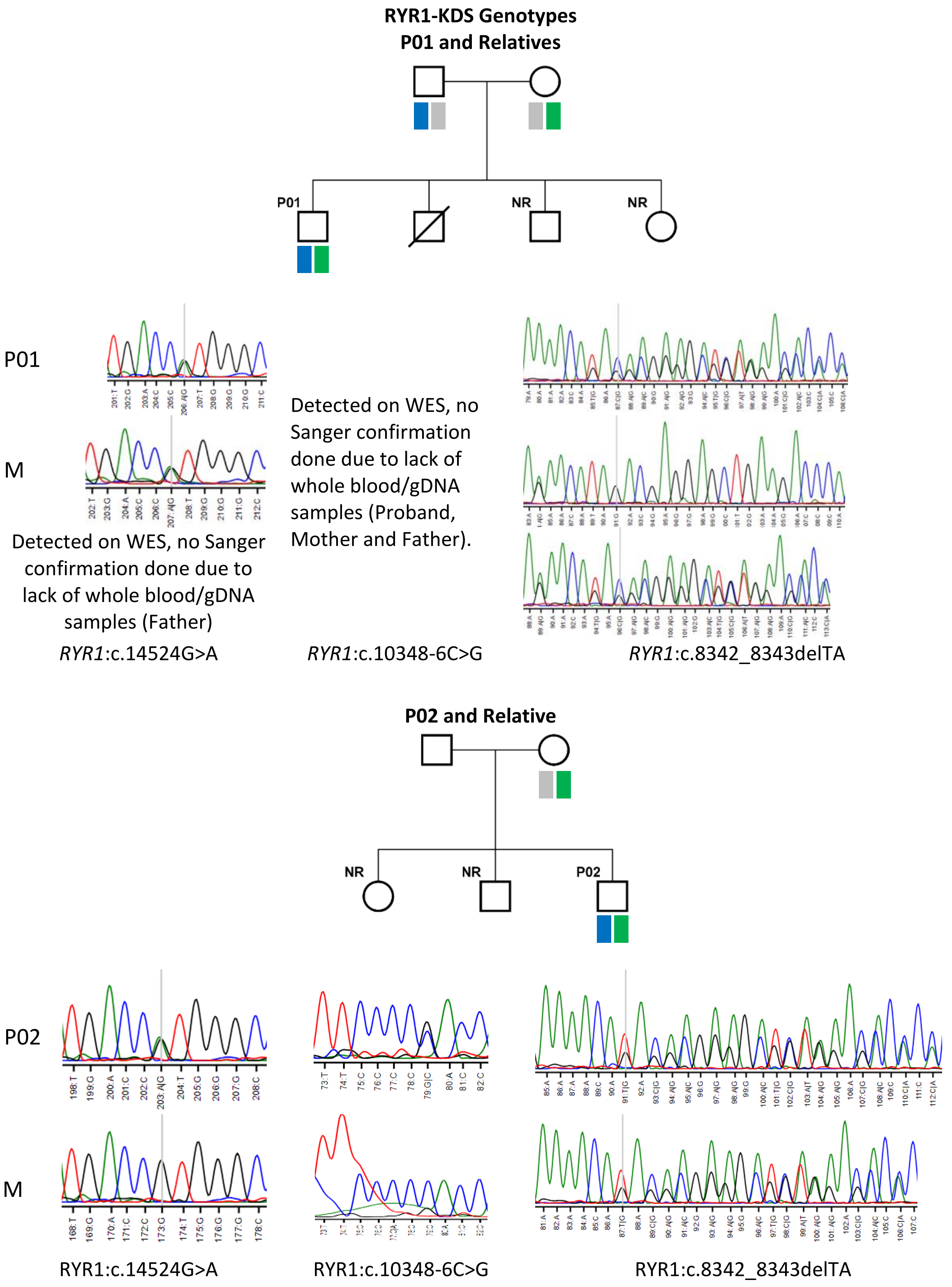
RYR1-KDS Genotypes
P01 and Relatives
P01
NR
NR
P01
M
Detected on WES, no Sanger confirmation done due to lack of whole blood/gDNA samples (Father)
Detected on WES, no Sanger confirmation done due to lack of whole blood/gDNA samples (Proband, Mother and Father).
RYR1:c.14524G>A
RYR1:c.10348-6C>G
RYR1:c.8342_8343delTA
P02 and Relative
NR
NR
P02
P02
M
RYR1:c.14524G>A
RYR1:c.10348-6C>G
RYR1:c.8342_8343delTA

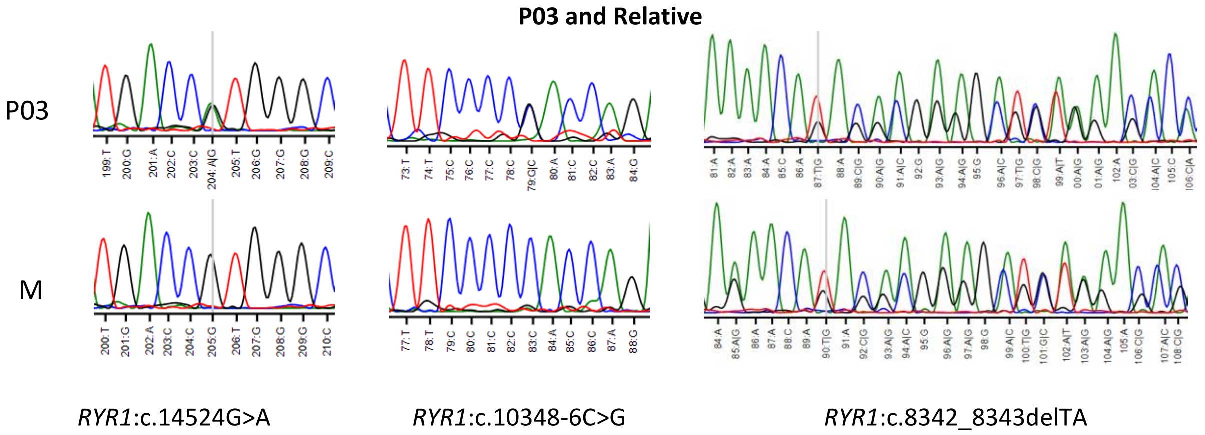

P03 and Relative
P03
M
RYR1:c.14524G>A
RYR1:c.10348-6C>G
RYR1:c.8342_8343delTA

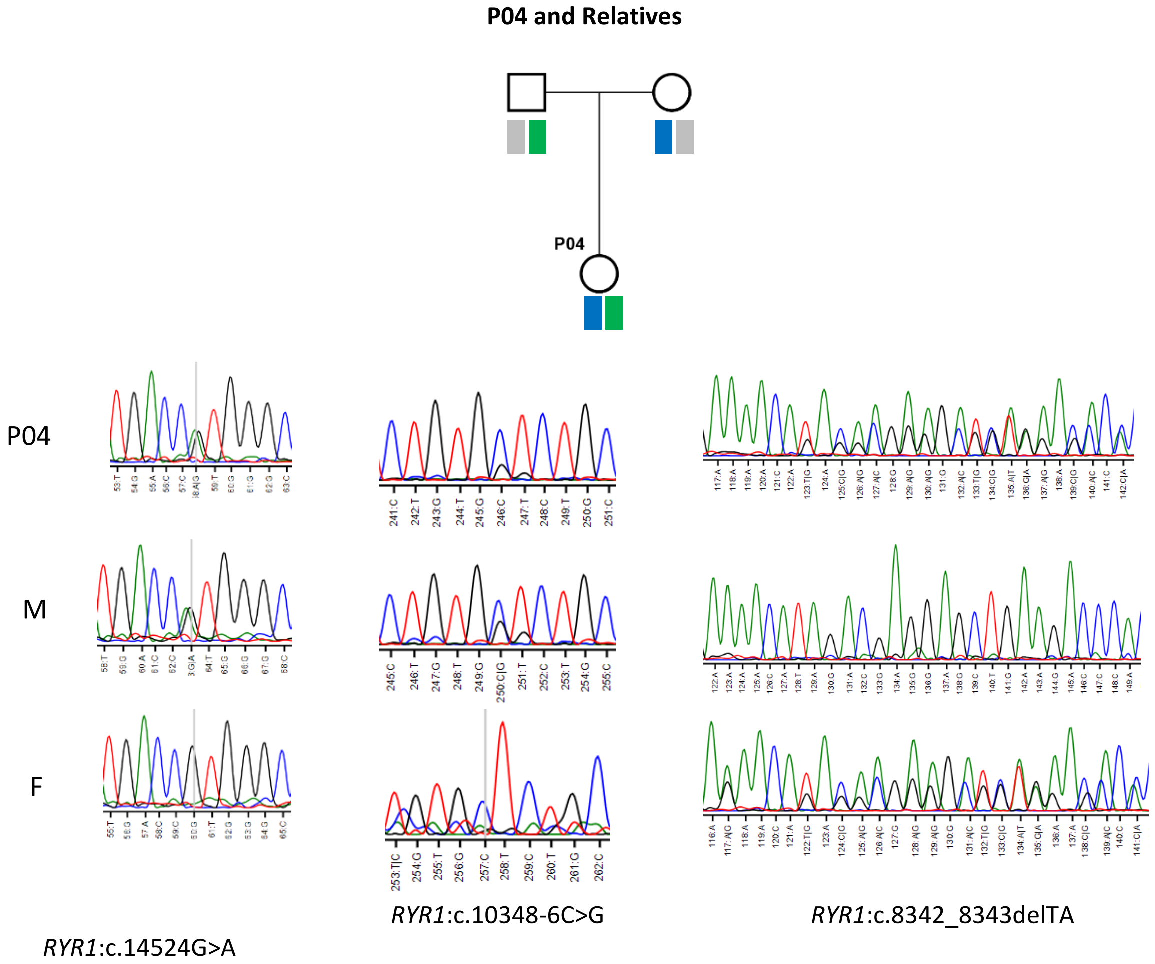

P04 and Relatives
P04
M
F
RYR1:c.14524G>A
RYR1:c.10348-6C>G
RYR1:c.8342_8343delTA

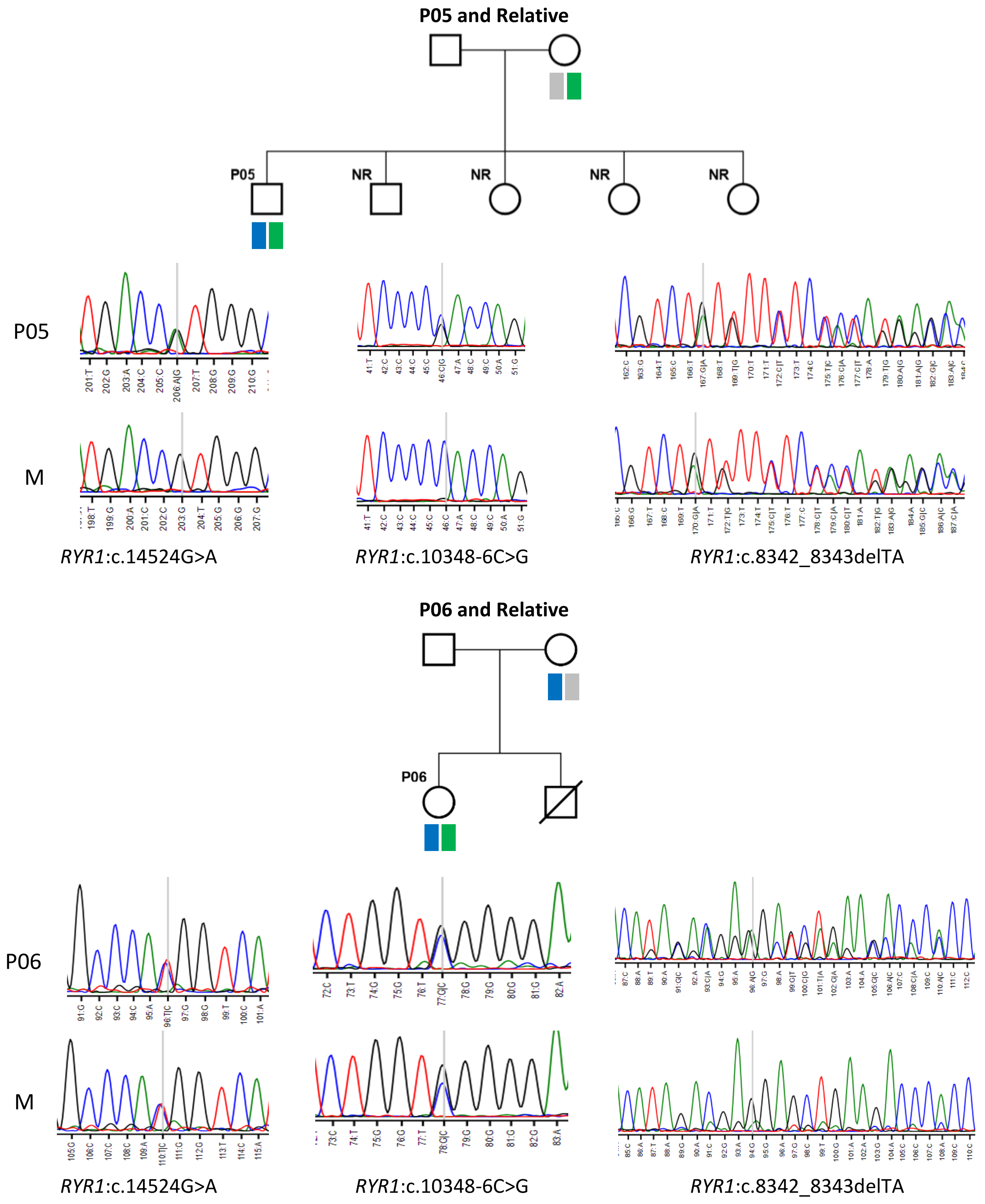
P05 and Relative
P05
NR
NR
NR
NR
P05
M
RYR1:c.14524G>A
RYR1:c.10348-6C>G
RYR1:c.8342_8343delTA
P06 and Relative
P06
P06
M
RYR1:c.14524G>A
RYR1:c.10348-6C>G
RYR1:c.8342_8343delTA

P07 and Relatives

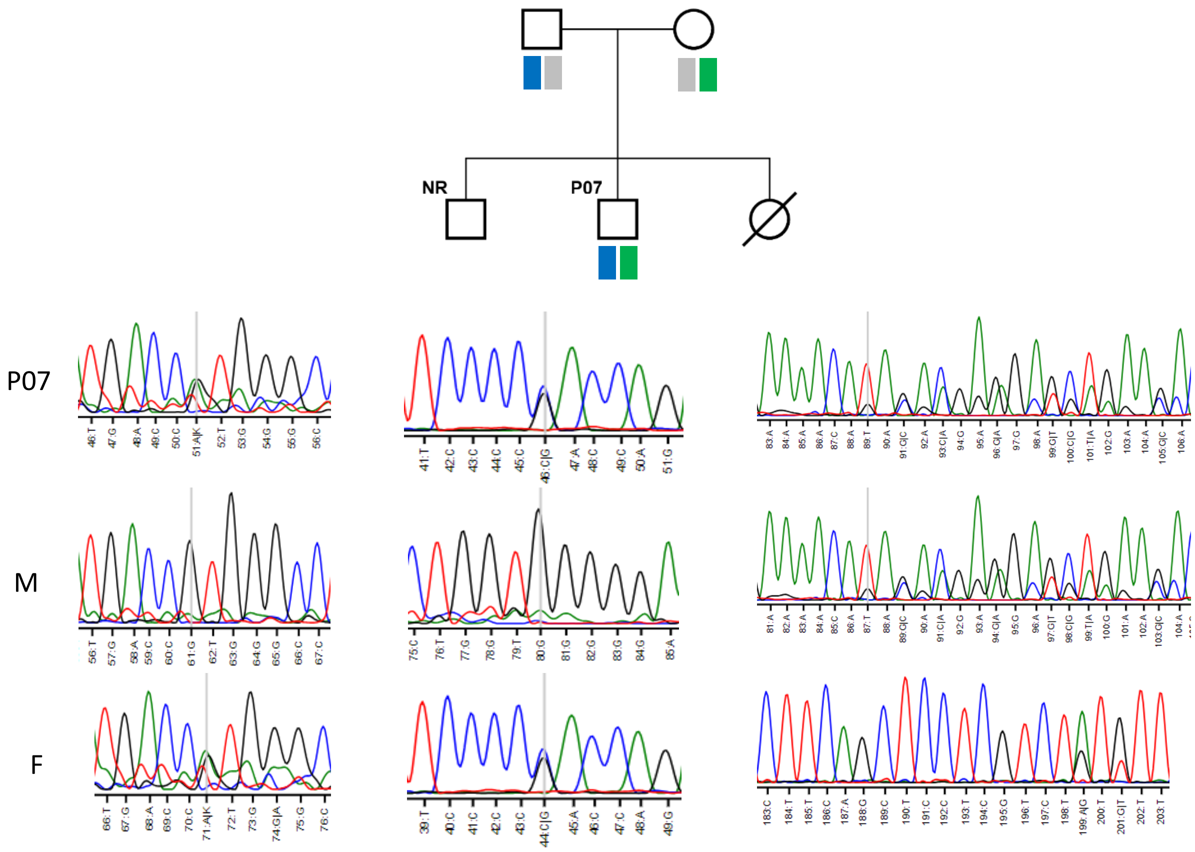

RYR1:c.14524G>A

RYR1:c.10348-6C>G

RYR1:c.8342_8343delTA

P08 and Relative

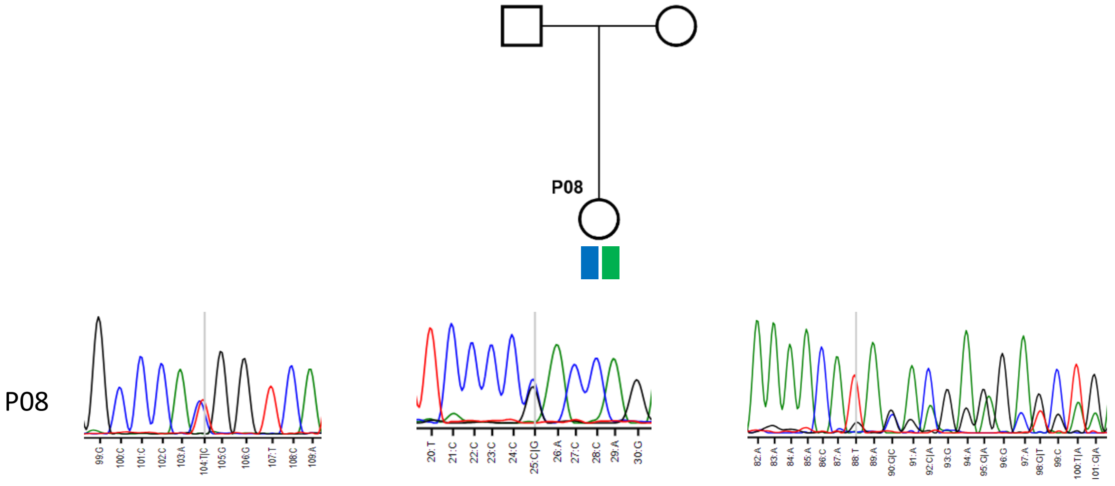

RYR1:c.14524G>A
Detected on WES, no Sanger confirmation done due to lack of whole blood/gDNA samples (Mother and Father)

RYR1:c.10348-6C>G
Detected on WES, no Sanger confirmation done due to lack of whole blood/gDNA samples (Mother and Father)

RYR1:c.8342_8343delTA
Detected on WES, no Sanger confirmation done due to lack of whole blood/gDNA samples (Mother and Father)

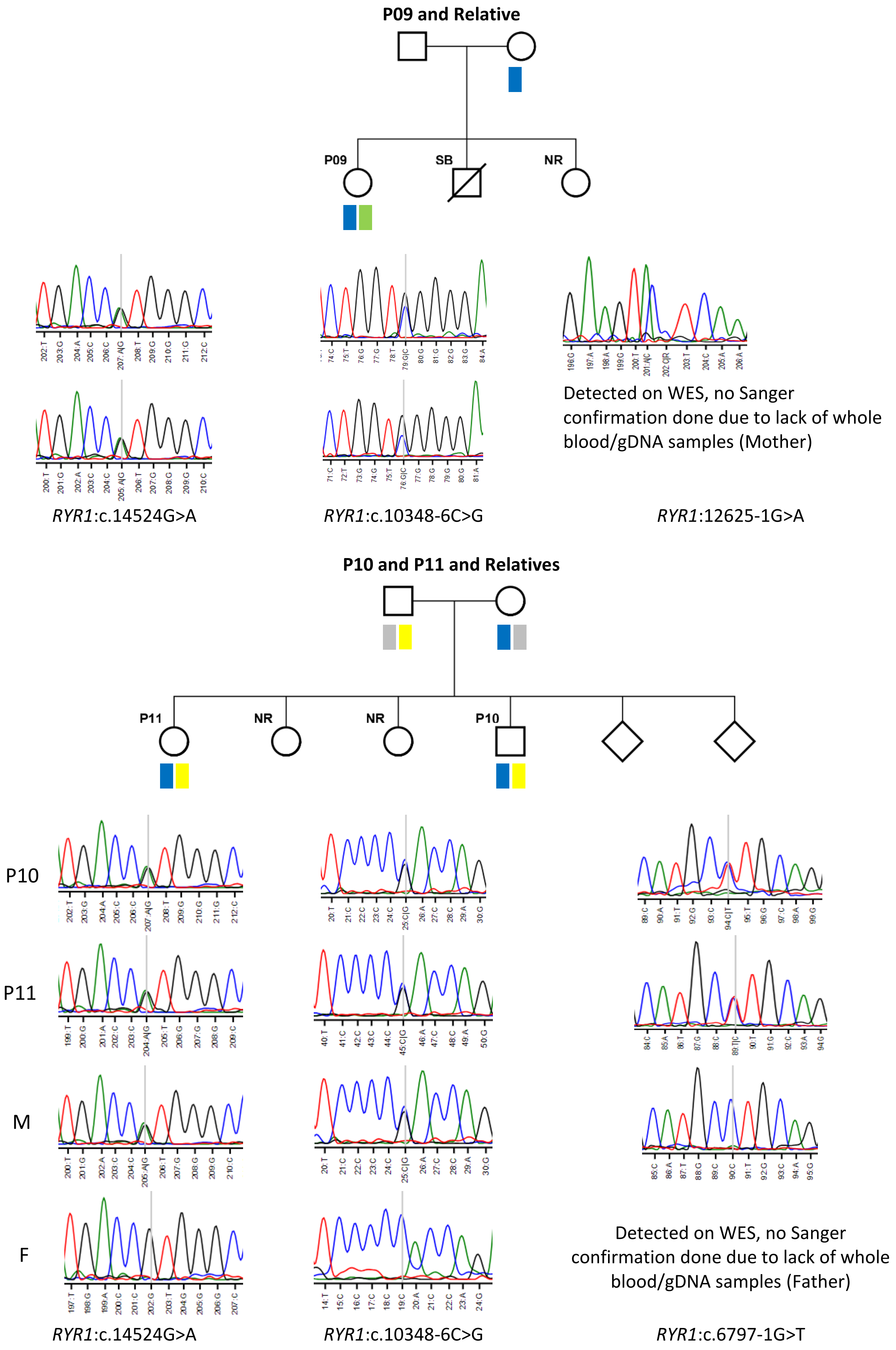

P09 and Relative
P09
SB
NR
Detected on WES, no Sanger confirmation done due to lack of whole blood/gDNA samples (Mother)
RYR1:c.14524G>A
RYR1:c.10348-6C>G
RYR1:12625-1G>A
P10 and P11 and Relatives
P11
NR
NR
P10
P10
P11
M
F
Detected on WES, no Sanger confirmation done due to lack of whole blood/gDNA samples (Father)
RYR1:c.14524G>A
RYR1:c.10348-6C>G
RYR1:c.6797-1G>T

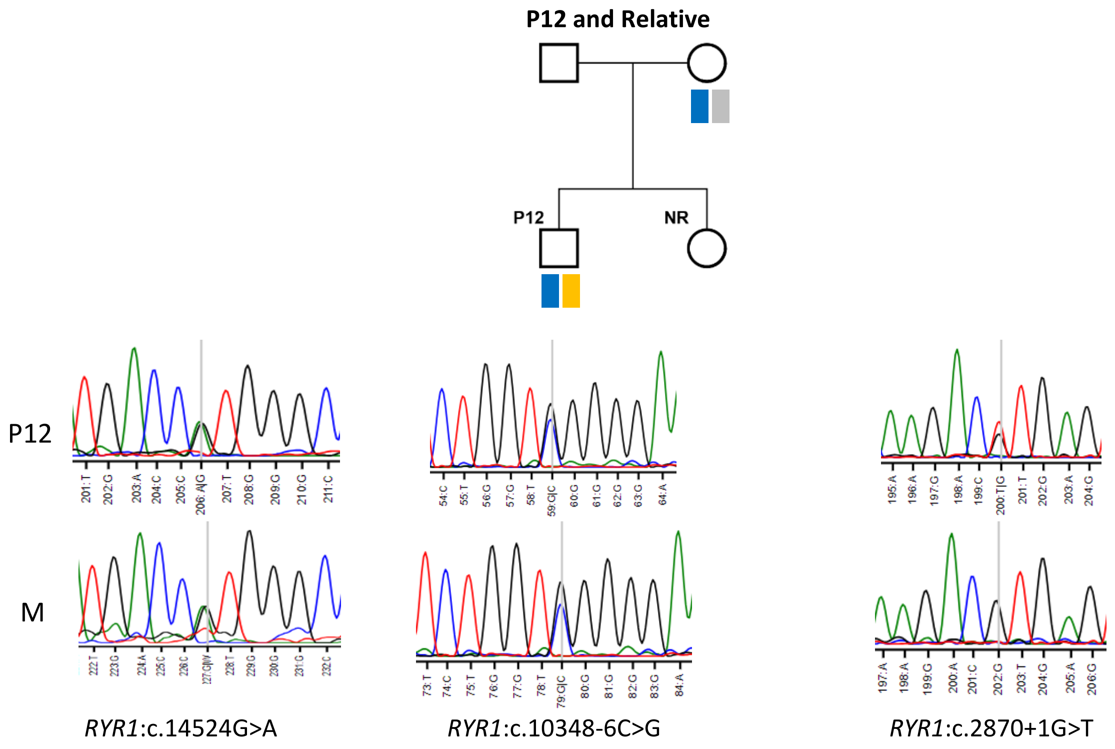
P12 and Relative
P12
NR
P12
M
RYR1:c.14524G>A
RYR1:c.10348-6C>G
RYR1:c.2870+1G>T

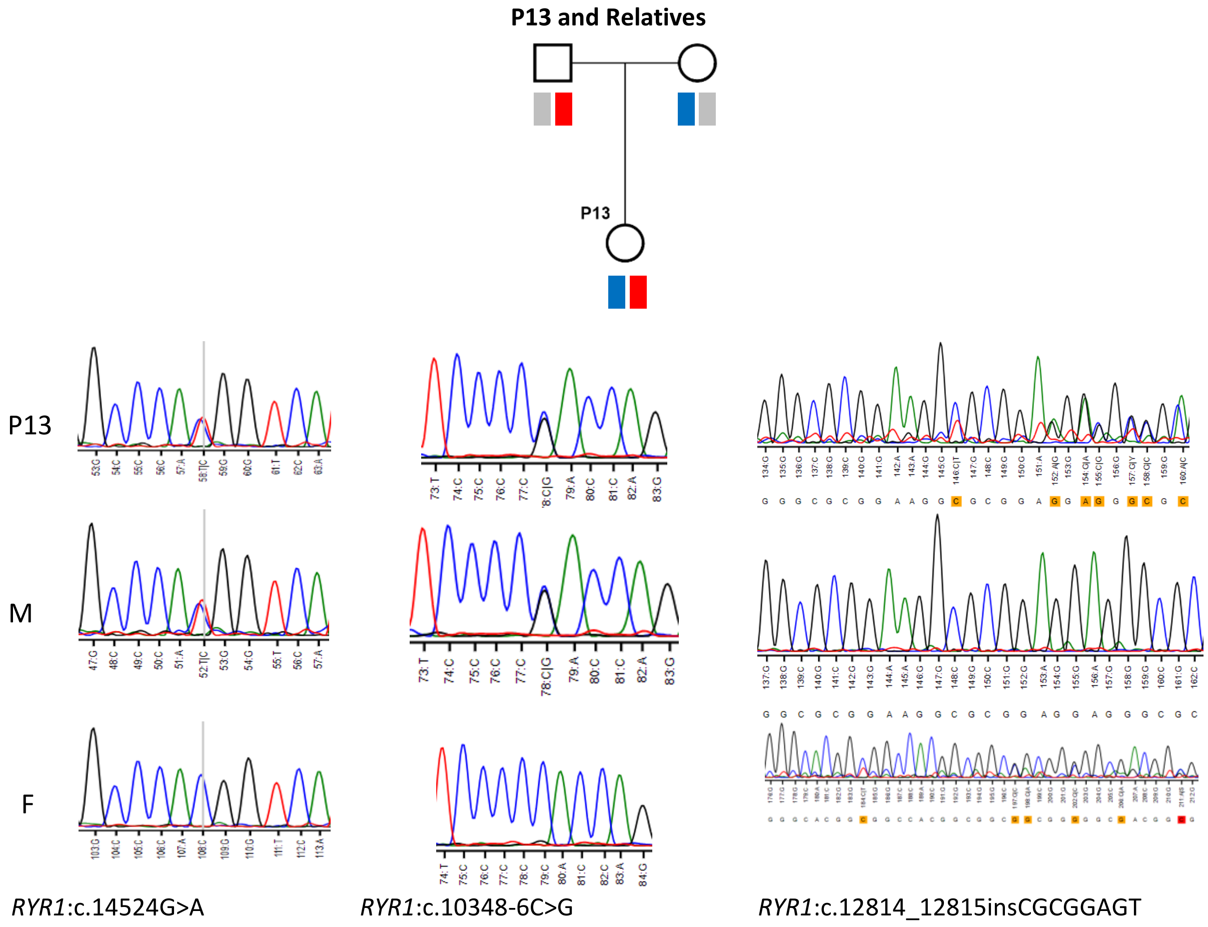
P13 and Relatives
P13
P13
M
F
RYR1:c.14524G>A
RYR1:c.10348-6C>G
RYR1:c.12814_12815insCGCGGAGT

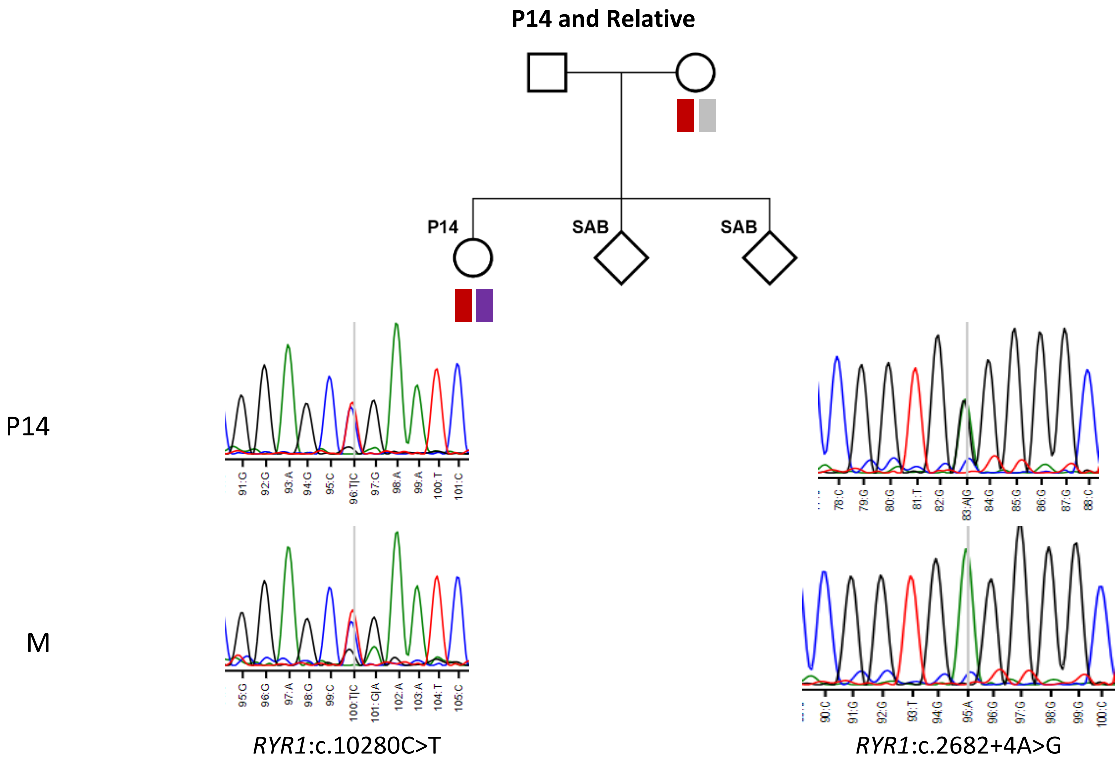
P14 and Relative
P14
SAB
SAB
P14
M
RYR1:c.10280C>T
RYR1:c.2682+4A>G

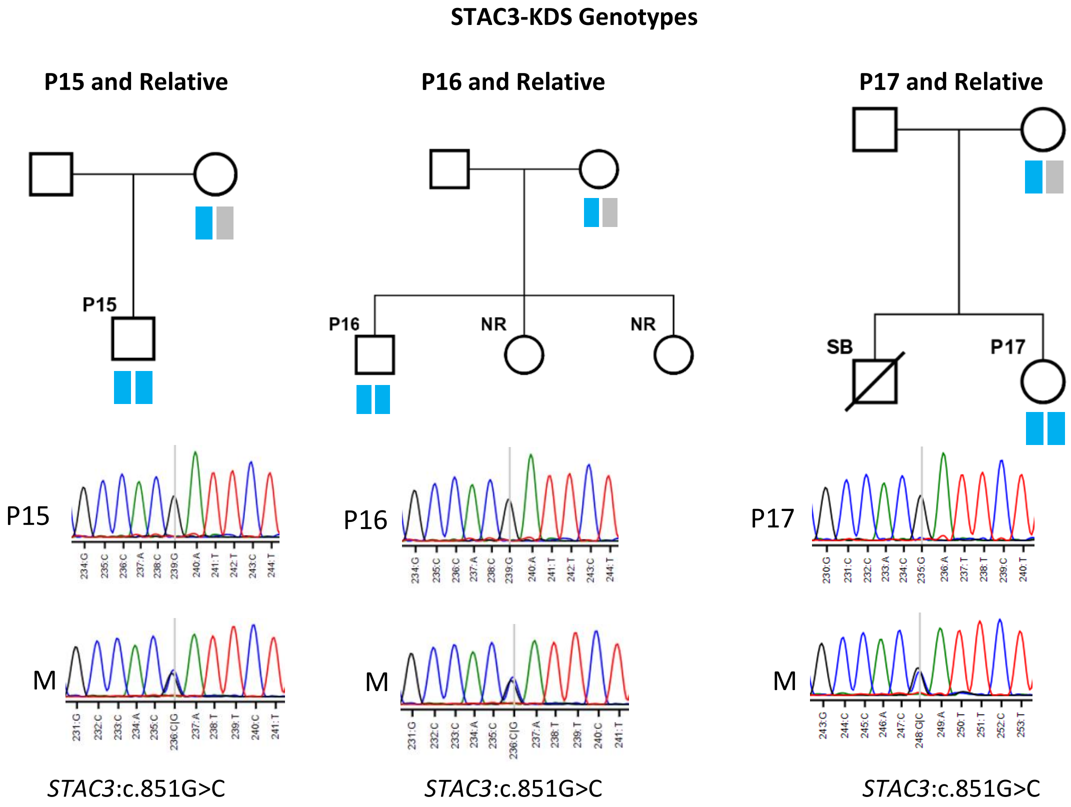
STAC3-KDS Genotypes
P15 and Relative
P16 and Relative
P17 and Relative
P15
P16
NR
NR
SB
P17
P15
M
P16
M
P17
M
STAC3:c.851G>C
STAC3:c.851G>C
STAC3:c.851G>C

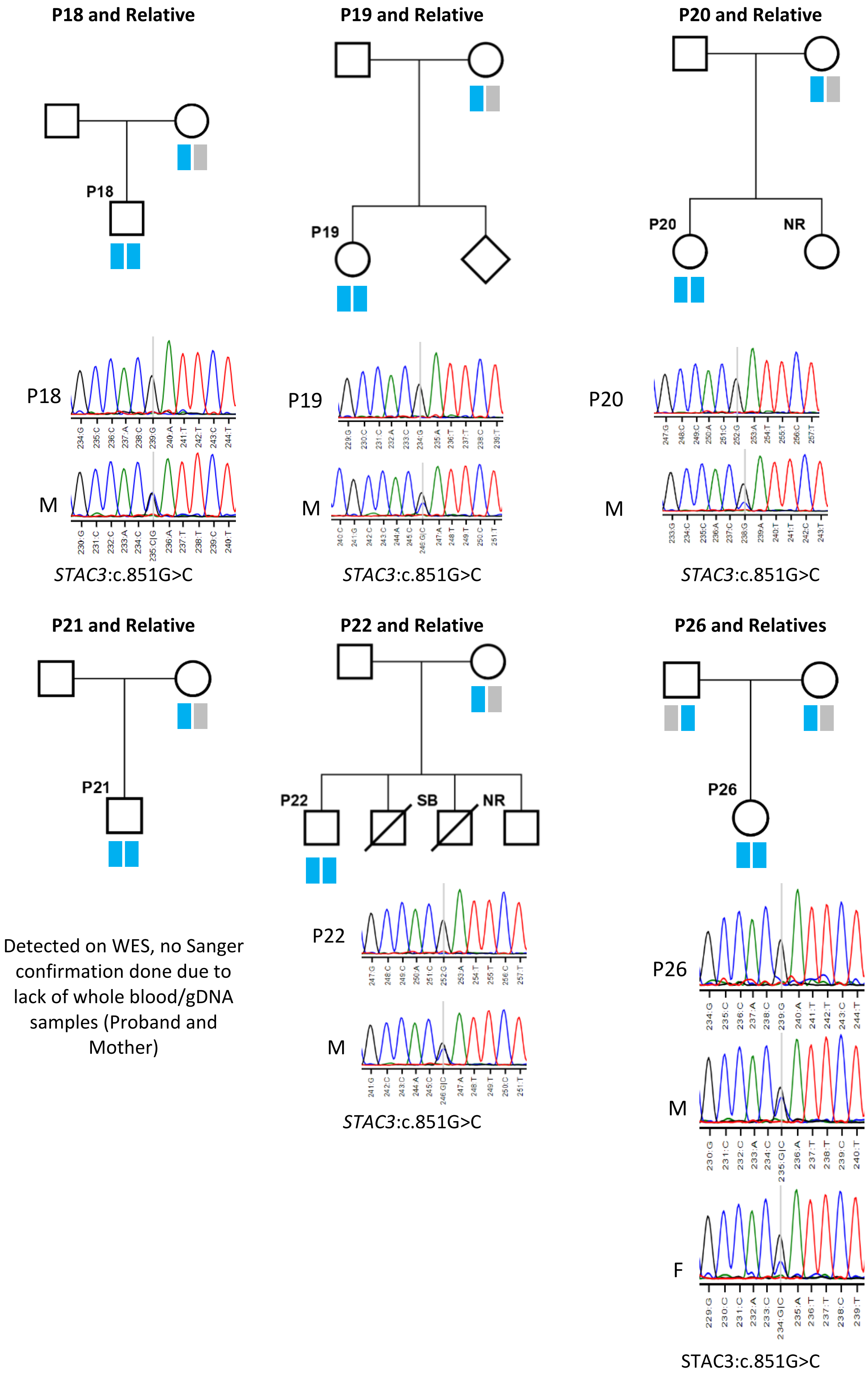
P18 and Relative
P18
P18
M
STAC3:c.851G>C
P19 and Relative
P19
P19
M
STAC3:c.851G>C
P20 and Relative
P20
NR
P20
M
STAC3:c.851G>C
P21 and Relative
P21
Detected on WES, no Sanger confirmation done due to lack of whole blood/gDNA samples (Proband and Mother)
P22 and Relative
P22
SB
NR
P22
M
STAC3:c.851G>C
P26 and Relatives
P26
P26
M
F
STAC3:c.851G>C

P23 and Relative

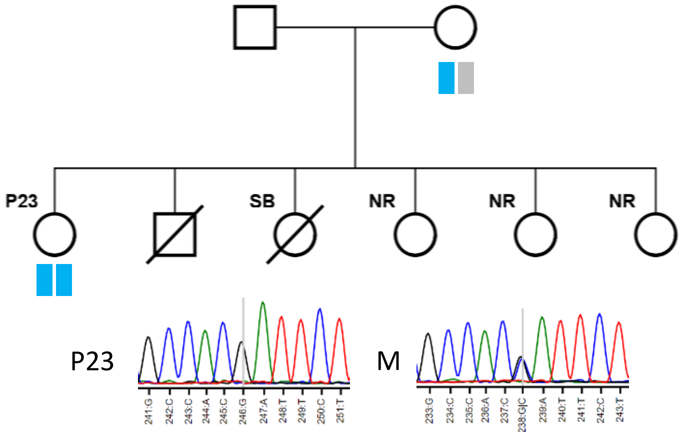

STAC3:c.851G>C

P24 and Relative

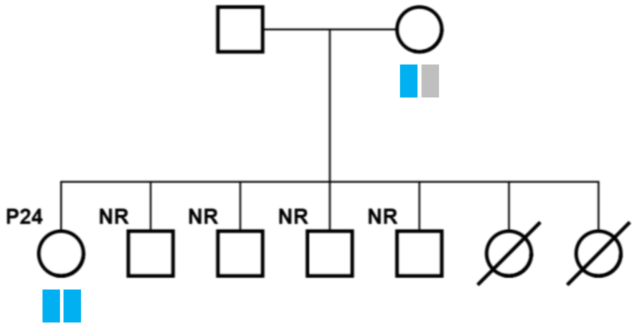

Detected on WES, no Sanger confirmation done due to lack of whole blood/gDNA samples (Proband and Mother)

P29 and Relative

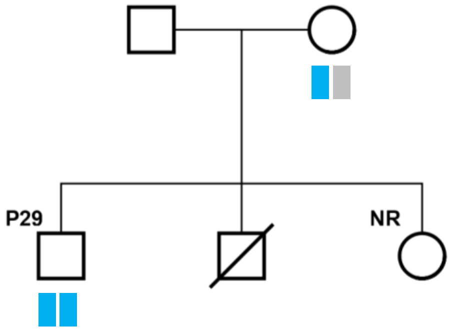

Detected on WES, no Sanger confirmation done due to lack of whole blood/gDNA samples (Proband and Mother)

P25 and Relative

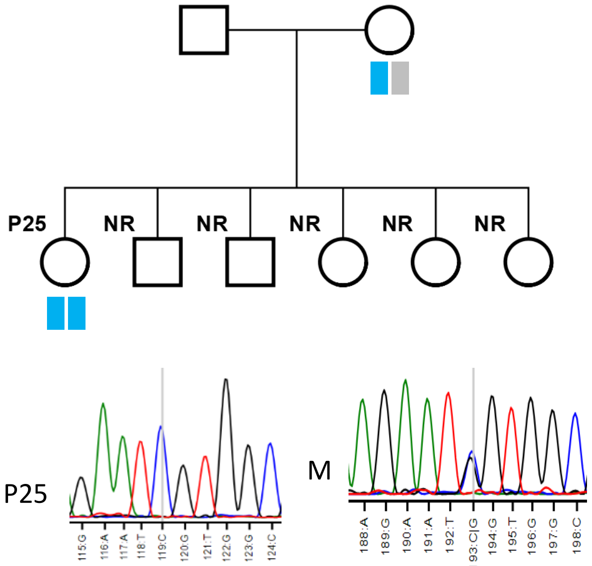

STAC3:c.851G>C

P30 and Relative

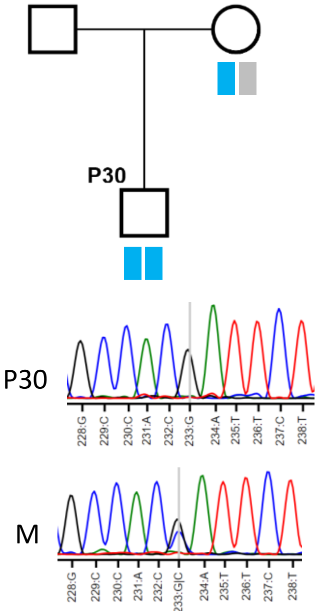

STAC3:c.851G>C

P34 and P27 and Relatives

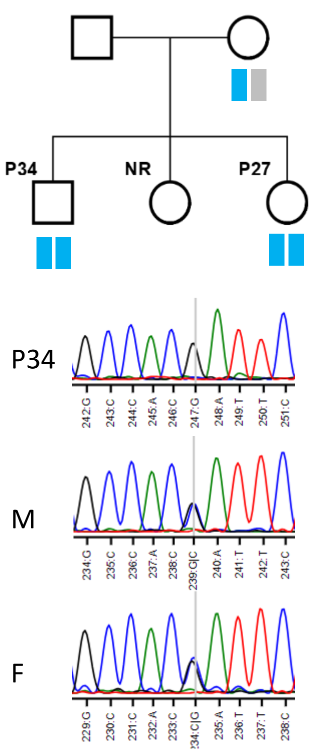

Detected on WES, no Sanger confirmation done due to lack of whole blood/gDNA samples (P27)

STAC3:c.851G>C

P28 and Relative

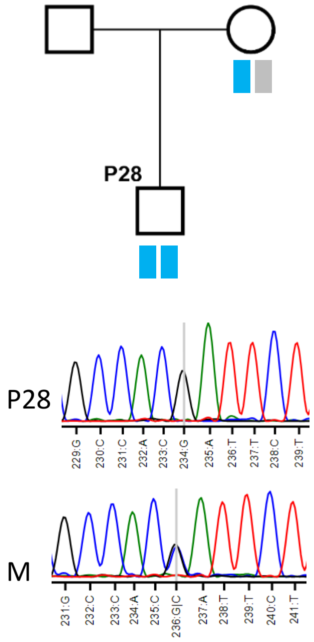

STAC3:c.851G>C

P31 and Relative

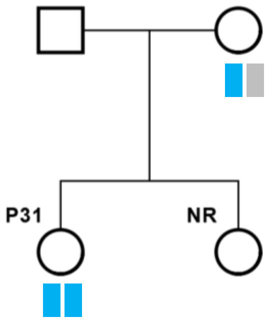

Detected on WES, no Sanger confirmation done due to lack of whole blood/gDNA samples (Proband and Mother)

P32 and Relative

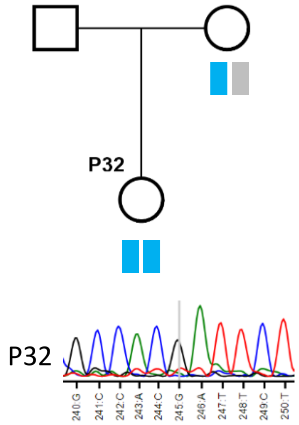

Detected on WES, no Sanger confirmation done due to lack of whole blood/gDNA samples (Mother).

STAC3:c.851G>C

P33 and Relative

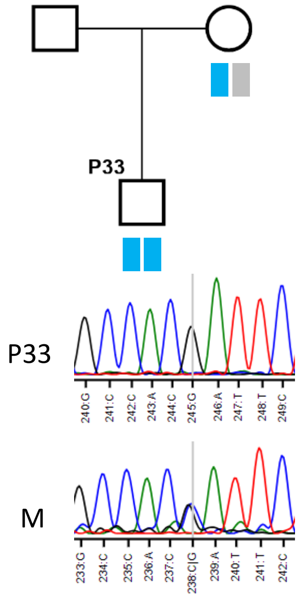

STAC3:c.851G>C

P35 and Relative

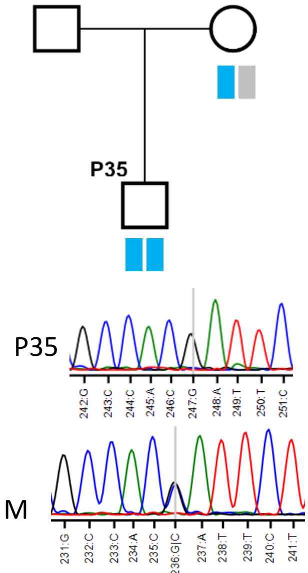

STAC3:c.851G>C

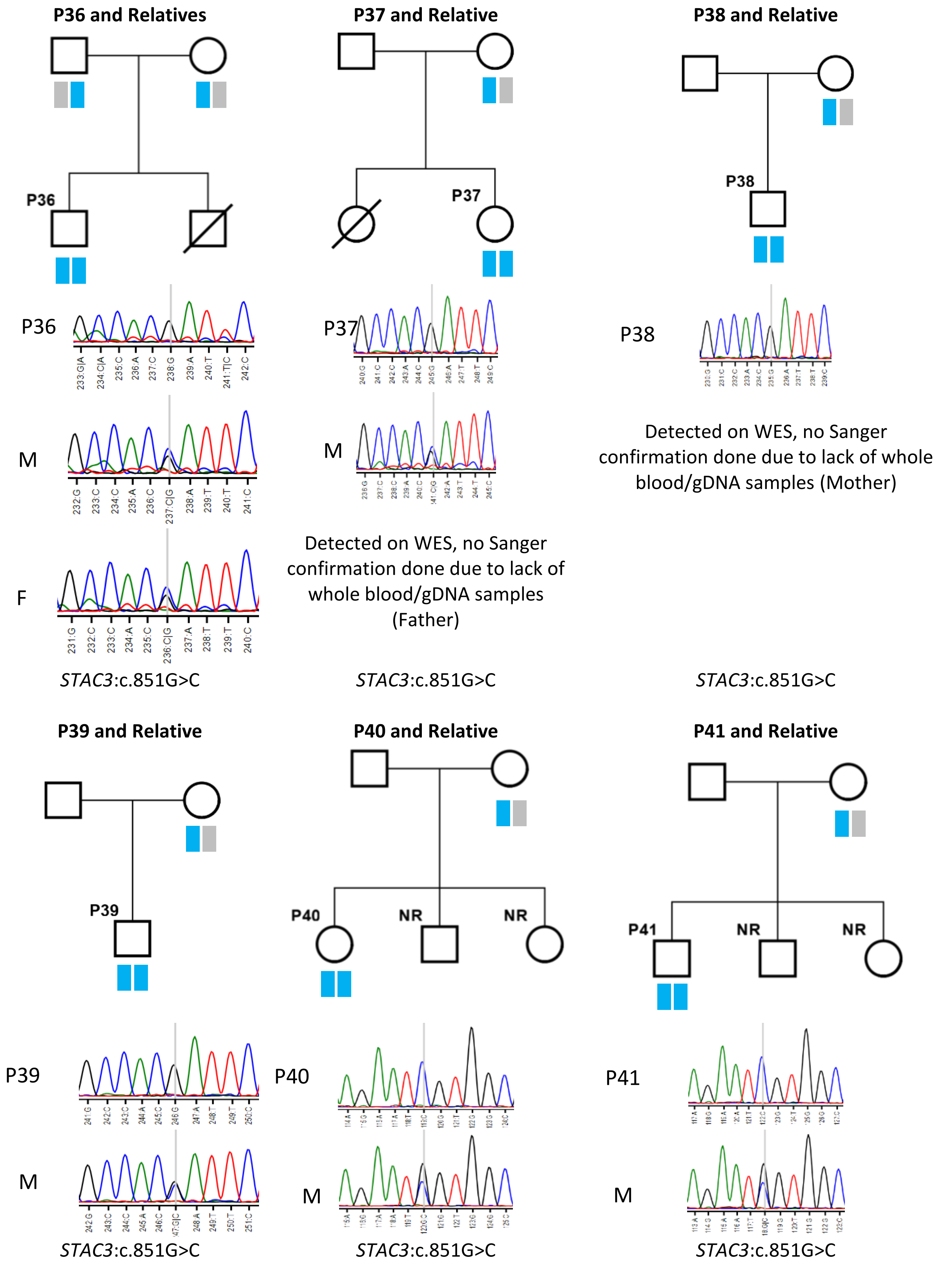
P36 and Relatives
P37 and Relative
P38 and Relative
P36
P37
P38
P36
M
F
P37
M
P38
Detected on WES, no Sanger confirmation done due to lack of whole blood/gDNA samples (Father)
Detected on WES, no Sanger confirmation done due to lack of whole blood/gDNA samples (Mother)
STAC3:c.851G>C
STAC3:c.851G>C
STAC3:c.851G>C
P39 and Relative
P40 and Relative
P41 and Relative
P39
P40
NR
NR
P41
NR
NR
P39
M
P40
M
P41
M
STAC3:c.851G>C
STAC3:c.851G>C
STAC3:c.851G>C

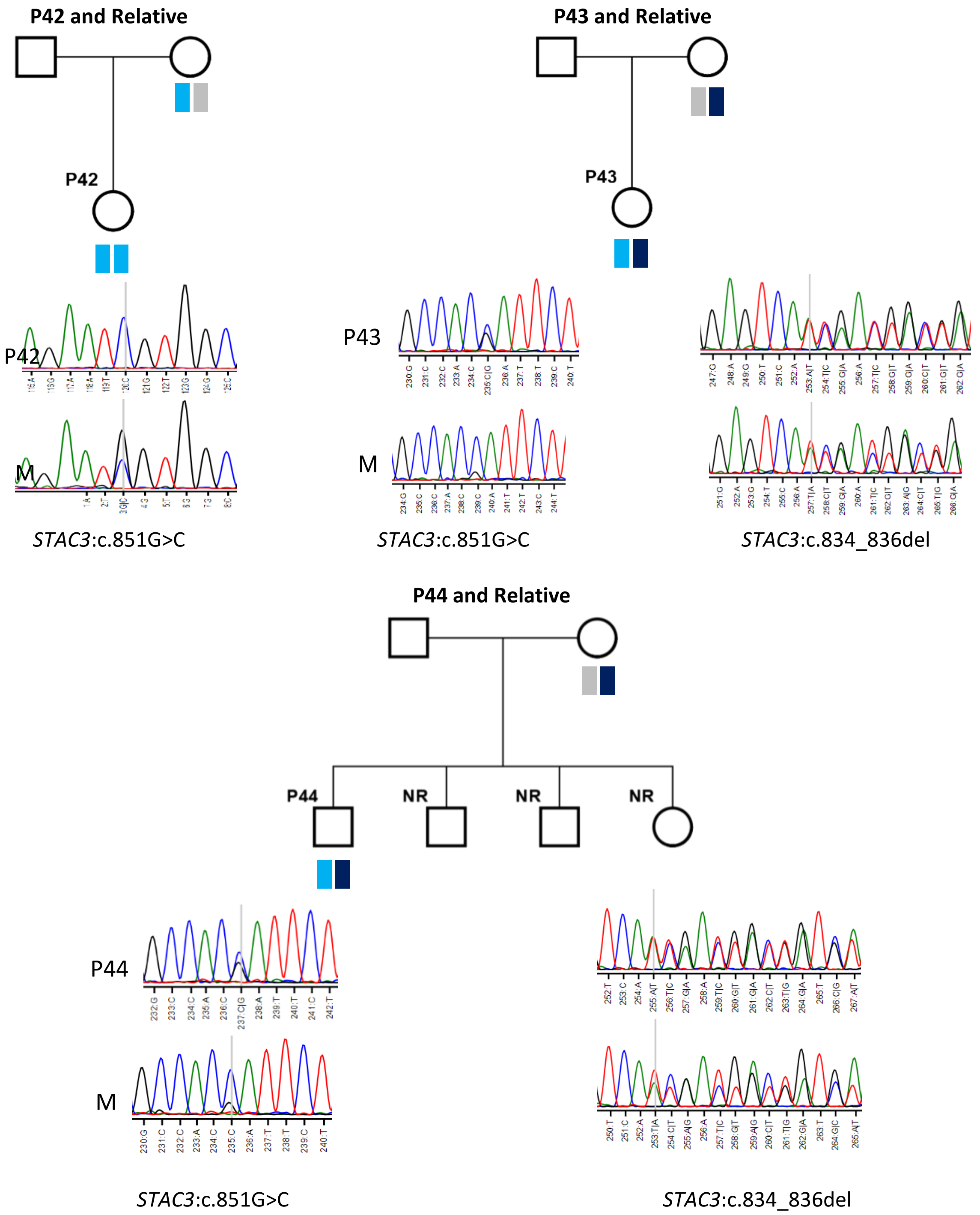

Abbreviations: NR, Not recruited; SB, Still birth; SAB, Spontaneous abortion

- Wildtype
- *RYR1*:[c.10348-6C>G, c.14524G>A]
- *RYR1*:c.8342_8343delTA
- *RYR1*:c.12625-1G>A
- *RYR1:c.2682+4A>G*
- *STAC3:c.834_836del*
- *RYR1*:c.6797-1G>T
- *RYR1*:c.2870+1G>T
- *RYR1*:c.12814_12815insCGCGGAGT
- *RYR1*:c.10280C>T
- *STAC3:c.851G>C*
